# Evaluation of impact of engaging federations of women groups to improve women’s nutrition interventions- before, during and after pregnancy in social and economically backward geographies: Evidence from three eastern Indian States

**DOI:** 10.1371/journal.pone.0291866

**Published:** 2023-10-05

**Authors:** Abhishek Kumar, Vani Sethi, Arjan de Wagt, Rabi N. Parhi, Sourav Bhattacharjee, Sayeed Unisa, Reshmi R. S., Abhishek Saraswat, Nita Kejrewal, Monica Shrivastava, Lopamudra Tripathy, Zivai Murira, Sheila Vir, on behalf of the Swabhimaan Study Group

**Affiliations:** 1 Institute of Economic Growth, Delhi University Enclave (North Campus), Delhi, India; 2 United Nations Children’s Fund Regional Office for South Asia, Kathmandu, Nepal; 3 United Nations Children’s Fund, India Country Office, New Delhi, India; 4 United Nations Children’s Fund, Bihar, India; 5 United Nations Children’s Fund, Odisha, India; 6 International Institute for Population Sciences, Mumbai, India; 7 National Rural Livelihood Mission, Ministry of Rural Development, Government of India, New Delhi, India; 8 ROSHNI-Centre of Women Collectives led Social Action, New Delhi, India; 9 Public Health Nutrition and Development Centre, New Delhi, India; University of Hyderabad, INDIA

## Abstract

**Background:**

Undernutrition–before, during and after pregnancy endangers the health and well-being of the mother and contributes to sub-optimal fetal development and growth. A non-randomized controlled evaluation was undertaken to assess the impact of engaging federations of women’s group on coverage of nutrition interventions and on nutrition status of women in the designated poverty pockets of three Indian states—Bihar, Chhattisgarh, and Odisha.

**Method:**

The impact evaluation is based on two rounds of cross-sectional data from 5 resource poor blocks across 3 States, assigning 162 villages to the intervention arm and 151 villages to the control arm. The cross-sectional baseline (2016–17) and endline survey (2021–22) covered a total of 10491 adolescent girls (10–19 years), 4271 pregnant women (15–49 years) and 13521 mothers of children under age two years (15–49 years). Exposure was defined based on participation in the participatory learning and action meetings, and fixed monthly health camps (Adolescent Health Days (AHDs) and Village Health Sanitation and Nutrition Days (VHSNDs)). Logistic regression models were applied to establish the association between exposure to programme activities and improvement in coverage of nutrition interventions and outcomes.

**Results:**

In the intervention area at endline, 27–38% of women participated in the participatory learning and action meetings organized by women’s groups. Pregnant women participating in programme activities were two times more likely to receive an antenatal care visit in the first trimester of pregnancy (Odds ratio: 2.55 95% CI-1.68–3.88), while mothers of children under 2 were 60% more likely to receive 4 ANC visits (Odds ratio: 1.61, 95% CI- 1.30–2.02). Odds of consuming a diversified diet was higher among both pregnant women (Odds ratio: 2.05, 95% CI- 1.41–2.99) and mother of children under 2 years of age (Odds ratio: 1.38, 95% CI- 1.08–1.77) among those participating in programme activities in the intervention arm. Access to commodities for WASH including safe sanitation services (Odds ratio: 1.80, 95% CI- 1.38–2.36) and sanitary pads (Odds ratio: 1.64, 95% CI- 1.20–2.22) was higher among adolescent girls participating in programme activities.

**Conclusion:**

Women’s groups led participatory learning and action approaches coupled with strengthening of the supply side delivery mechanisms resulted in higher coverage of health and nutrition services. However, we found that frequency of participation was low and there was limited impact on the nutritional outcomes. Therefore, higher frequency of participation in programme activities is recommended to modify behaviour and achieve quick gains in nutritional outcomes.

## 1. Introduction

Women’s nutrition, before and during pregnancy have important implications for fetal growth and well-being of the mother [1, 2]. Globally, poor nutrition in utero is accountable for 800000 neonatal deaths annually through small for gestational age births [2–4]. Moreover, 3 million child deaths annually are caused due to undernutrition [2]. Latest data show that undernutrition continues to remain a major public health problem in India [5]. Recent estimates based on fifth round of National Family Health Survey reveals that Indian women enter pregnancy too young, thin and with poor nutrition [5]. Specifically, 23 per cent of women in reproductive age are too thin for their height, more than 50 per cent among pregnant women are anaemic and eight per cent of pregnant women are adolescents [5]. India’s has in place the policies and programs to provide health and nutrition services to adolescent girls and pregnant and lactating women. However, the slow progress in reducing undernutrition demands innovative strategies to address the persistent problem of poor nutritional status among women.

Towards this end, integrating nutrition programmes for promoting health interventions and increasing service uptake through women-centric poverty alleviation programmes are considered to be a feasible approach in low-resource settings [6–10]. Notable examples from India where women’s collectives have been engaged and trained to undertake livelihood activities are Kudumbashree (Kerala), Society for Elimination of Rural Poverty Project (Andhra Pradesh and Telangana), Community Health Care Management Initiative (West Bengal), Jamkhed (Maharashtra), Urban Health Resource Centre (urban health models), Mahila Abhivrudhi Society (Andhra Pradesh). Similar food security programs have been implemented in Nepal (Suaahara), and Indonesia (community conditional cash transfer program) [11, 12]. Most of these programmes aimed to strengthen the health services delivery system in addition to intervening with community groups [13].

Despite these initiatives, women’s self-help groups (SHGs) and their federations supported by the Deendayal Antyodaya Yojana-National Rural Livelihoods Mission (DAY-NRLM), the Government of India’s flagship poverty alleviation programme, remain an underutilized platform for improving reach and use of essential nutrition interventions among women residing in income-constrained settings [14]. A 2016 scoping study by CARE and UNICEF India suggested that DAY-NRLM village organisations (VOs) have the potential to manage grants for improving last-mile delivery of essential nutrition services for women, provided they are enabled, supervised and incentivised [13, 15]. DAY-NRLM is one of the nodal agencies included in the implementation strategy of the Poshan Abhiyaan 2018 programme, the National Nutrition Mission of Government of India.

Capitalising on the NRLM platform, the Swabhimaan programme was launched in 2016 as a 5-year initiative to improve women’s nutrition before, during and after pregnancy. Swabhimaan is a comprehensive package of community-led interventions delivered by women of DAY-NRLM-supported by federations of SHGs comprising VOs and the higher order cluster-level federations (CLFs). The Swabhimaan programme aims to improve the nutrition status of women before, after and during pregnancy in three eastern states of India: Bihar, Chhattisgarh, and Odisha [16]. Notably, more than a quarter of women (15–49 years) are thin across these States and there are designated poverty areas that have poorer nutrition indicators and low outreach of government services [5].

There are a few studies [10, 17–22] that evaluate and document the impact of engaging women’s collectives to improve health and nutrition outcomes. However, the key indicators of these studies are limited primarily to birth outcomes, pregnancy related services, and maternal and child mortality. To our knowledge there are only a couple of studies [21, 23] which specifically evaluate the impact of engaging women’s collectives on access to nutrition interventions and on nutrition outcomes of women during and after pregnancy. However, the evidence base available is limited. Moreover, the findings vary depending upon the study design and the context.

Most poverty alleviation programmes have also not been systematically evaluated to assess the impact on women’s empowerment and nutrition outcomes. Therefore, it was hypothesized that behaviour change strategy using Participatory Learning and Action (PLA) methods along with strengthening of delivery of existing health, ICDS and WASH services will lead to greater utilization of nutrition services in the poverty designated areas or blocks and would also improve the nutrition outcomes. This study had two objectives: (1) describe the implementation of the key interventions of the Swabhimaan programme across intervention and control areas, (2) assess whether the programme has led to improvement in access to nutrition specific and nutrition sensitive interventions and nutritional outcomes. Furthermore, the financial cost of supporting the Swabhimaan programme across each State is also presented.

In addition, econometric analysis was conducted to assess the impact of participation of SHG women members in PLA meetings, monthly fixed health camps (Adolescent Health Days (AHDs) and Village Health Sanitation and Nutrition Days (VHSNDs) on utilization of nutrition services as well as nutritional outcomes.

## 2. Methods

### 2.1 Swabhimaan programme description

Over the intervention period Swabhimaan’s community-led interventions was expected to primarily lead to:

1. A 15% decline in proportion of adolescents and mothers of children under two with low BMI (<18.5 kg/m^2^).
2. A 0.4 cm increment in mean mid-upper arm circumference (MUAC) among pregnant women.

The secondary outcome hypothesised an improvement of 5–20% in the coverage of 18 key nutrition-specific and nutrition-sensitive interventions (indicators stated in S2 Table).

The Swabhimaan programme strategy aims to improve the nutritional outcome by improving the coverage of following five nutritional interventions pertaining to:

- Improve food and nutrient intake of adolescent girls and women.
- Prevent micronutrient deficiencies and nutritional anaemia in girls and women.
- Increase access to preventive and development services provided by government during monthly Village Health Sanitation and Nutrition Days (VHSNDs) organized at village level by different government stakeholders (comprising of multi-purpose worker female (MPW-F/Auxiliary Nurse and Midwife), Accredited Social Health Activist (ASHA), Anganwadi Worker (AWW) and Anganwadi Helper (AWH), representatives of Department of Rural Development and Panchayati Raj) of the community and provide special care to nutritionally ‘at risk’ women, defined as those with mid-upper arm circumference (MUAC <23 cm),
- Increase access to education about water, sanitation, and hygiene (WASH) and access to infrastructure that supports WASH practices
- Prevent early, poorly spaced, and repeated pregnancies.

To assess the impact of the programme on the target groups (adolescent girls and women), the Swabhimaan programme design consisted of intervention and control areas. The State Rural Livelihoods Mission (SRLMs) of the three states were anchoring and implementing the Swabhimaan programme using their existing available administrative and human resource structure in coordination with additional technical and financial support from UNICEF. In the intervention areas, the programme was delivered through a combination of ***community- and systems-led actions***, while control areas received only systems strengthening actions. Community-led interventions were delivered through trained community cadres of SRLM who are members of VOs, namely Poshan Sakhis (literally meaning Nutrition Friends) or Community Resource Persons (CRPs) and Krishi Mitras (literally meaning Agriculture Friends) or Village Resource Persons (VRPs). These two community cadres are, as per the DAY-NRLM and SRLM system, part of the implementation structure. In Bihar, a separate cadre of Kishori Sakhis (literally meaning Adolescent Girls’ Friend) was created for reaching out and serving adolescent girls. While in Chhattisgarh and Odisha, the Poshan Sakhi conducted the PLA meetings for adolescent girls.

As per the Swabhimaan strategy, Poshan Sakhis and Kishori Sakhis underwent three days of pre-service training on integrated nutrition microplanning. The training sessions included both theoretical orientation and practical aspects. The community cadres were trained on consultative identification and prioritization of nutrition and related issues in their villages. A Participatory Learning and Action (PLA) technique was utilized for this purpose. They also learned how to develop an annual plan of activities with a budget to address these issues. Furthermore, the training covered the use of MUAC tapes and how to record and interpret MUAC measurements for screening nutritionally at-risk adolescent girls and women. Post-training, these two community cadres co-facilitated in the development of the integrated nutrition microplan with the block coordinator/ supervisor through a 12-day process, which could also spread over almost two months. Following this, Poshan Sakhis and Kishori Sakhis were trained over three days on the use of PLA technique to facilitate in the holding of monthly women’s group (Maitri Baithak) and adolescent girls’ group (Kishori Baithak) meetings and lead activities in their village/s as per the poshan microplan. More cost-intensive and complex grant management activities were led at the Federation level by CLFs. CRPs, Kishori Sakhi and CLFs received financial incentives for developing the poshan microplan. While Village Resource Persons (VRPs) at the Village Organisation level facilitated interventions with women farmers such as meeting on nutrition-sensitive agriculture practices and participatory learning and action along with setting up home-based nutrition gardens/backyard poultry.

The system strengthening activities which both intervention and control sites received included the following five components:

1. Strengthening VHSNDs to improve access to antenatal care, family planning and micronutrient supplementation through quarterly trainings of health service providers (AWW, ASHAs and ANMs), monthly review of nutrition indicators (such as height and weight), and the identification of women at risk of undernutrition (MUAC <23cm) for special supplementary food provision and counselling;
2. Strengthening Adolescent Health Days (AHD) to improve access to adolescent health and nutrition services (S2 Table) via quarterly trainings of health service providers;
3. Organising an extended VHSND once every six months for newlyweds and women, including individual counselling and information about entitlement camps;
4. Holding annual training and follow-up meetings with service providers from department of Food and Civil supplies, Integrated Child Development Services (ICDS), water and sanitation departments to help them improve the delivery of entitlements and services;
5. Ensuring regular review meetings are held with representation across government departments involved in service delivery.

Therefore, in addition to DAY-NRLM, system strengthening activities engaged four other government departments: a) Department of Health and Family Welfare for VHSND strengthening, b) Department of Women and Child Development for increasing ICDS reach and quality, c) Department of Drinking Water and Sanitation for improving water quality and achieving open defecation free villages and districts, and d) Department of Food Civil Supplies and Consumer Affairs for increasing coverage of food subsidy schemes under the Public Distribution System.

In each state, the process monitoring and progress review was done through project management units at State, district, and block levels (SPMU, DPMU, BPMU). Additionally, in the Swabhimaan Programme areas, CRPs/ Poshan Sakhis, developed poshan microplan, mobilize women and lead activities as per poshan microplan. The CRPs also collected data and reported coverage of community-led interventions on monthly monitoring formats which were available in both web-based and paper formats. The financing structure varied across States. Of the three states, Bihar state acted as the demonstration and learning site with its SRLM, as the programme activities and additional human resources were supported by UNICEF India. SRLM programme unit structures at State, District and Block level as well as community institutions i.e., CLFs, VOs and SHGs were leveraged to undertake the community interventions programme, process monitoring and progress review along with routine programme system activities. In Bihar, additional positions were supported by UNICEF at State, block, cluster federation levels and village organization levels (SPMU, BPMU, CLF and VO). In addition, a separate trained community resource persons (CRPs) (Kishori Sakhi) at VO levels in Bihar, additional to SRLM structure, contributed in reaching out to adolescent girls. In Chhattisgarh and Odisha, the initiative was led by the respective SRLMs through their existing structures, human resources, and cadres (no additional CRPs) from SHG to State level, while UNICEF only provided the technical assistance at State and Block level. Here, UNICEF to SRLM invested cost ratio was at 1:5. In all three States, UNICEF and SRLMs partnered with relevant non-government partners (and resource technical experts) to develop the required capacity building tools and methods, convergence with line departments and with academic partners for the impact and process evaluation components of the programme.

The total cost of implementing the Swabhimaan programme for the period (2016–20) was USD 5,327,751 of which USD 3,599,240 (70%) was transferred to SRLM by UNICEF while rest USD 1,728,511 was used by UNICEF for capacity building, research activities and programme evaluation (S1 Table). Costing analysis was done alongside this study for Bihar. The total cost funded by UNICEF for implementing the programme in Bihar was USD 433,688 which was 25% of the total UNICEF budget support (USD 1,728,511) to the entire programme and less than 10% of the total UNICEF and SRLM budget support (USD 53,27,751) to the programme across all the three States. From 2018 onwards, the programme continues and has scaled-up from 4 to 10 districts with increase in budget contributions of SRLM and UNICEF.

### 2.2 Target groups

The Swabhimaan programme impact evaluation was undertaken in 4 Districts and 5 Blocks of the three project states of Bihar, Chhattisgarh, and Odisha. The selected areas have a high burden of undernutrition, with significant populations of scheduled caste and tribal groups [15]. Agriculture is the primary occupation and farmers rely heavily on rain-fed farming. In addition, these areas are designated as poverty blocks, with low literacy rates and limited awareness which contribute to excessive prevalence of undernutrition [15]. The primary target groups for the programme were adolescent girls aged 10–19 years, pregnant women, and mothers of children under 2 years of age. Secondary target groups included, ANMs, ASHAs, AWWs, mothers-in-law and husbands. Earlier studies have indicated that care practices during pregnancy are sub-optimal in these regions due to lack of awareness, cultural beliefs, and limited women’s involvement in decision making [15, 24].

### 2.3 Evaluation design

The Swabhimaan evaluation is a prospective, non-randomised controlled study (S1 Fig). Since 2017, across Bihar, Odisha and Chhattisgarh, five sites covering 162 villages constituting the intervention arm that delivered community-led interventions through VOs and CLFs. The control arm comprised 151 villages. Both intervention and control areas were in the resource poor blocks identified by DAY-NRLM in the four project districts: Purnia in Bihar, Angul and Koraput in Odisha and Bastar in Chhattisgarh. In Bihar, the demonstration site, 42 villages were allocated to the intervention and 31 villages to control arm located in Purnia district (Jalalgarh and Kasba blocks). In Odisha, 80 villages in Koraput block in the Koraput district and Pallahara block in the Angul district served as intervention areas, and 80 more served as control. In Bastar district, Chhattisgarh, 40 villages in Bastar block and 40 villages in Bakawand block were allocated to intervention and control arms. The unit of assignment to intervention and control was a gram panchayat (administrative units of around 5000 population) in Odisha and a cluster of villages in Bihar and Chhattisgarh, which was in alignment with NRLM identified administrative boundaries for managing the livelihoods programme of SHGs in these states.

### 2.4 Impact evaluation

The baseline and endline survey were carried out during 2016–17 and 2021–22 respectively. In Bihar, the baseline and endline surveys were carried out in the Jalalgarh and Kasba blocks of Purnea district in 2016 and 2021, respectively. In Chhattisgarh, the surveys took place in the Bastar block in 2017 for the baseline and in 2021–22 for the endline. In Odisha, the baseline and endline survey were conducted in Koraput block, Koraput district and in Pallahara block in Angul district in 2016–17 and 2021 respectively. The survey followed a cross-sectional design that covered adolescent girls, pregnant women, and mothers of children under two years across the three states–Bihar, Chhattisgarh and Odisha, covering both intervention and control areas:

- Bihar: 23 intervention and 22 control areas
- Odisha: 41 intervention and 48 control areas
- Chhattisgarh: 21 intervention and 19 control areas

### 2.5 Sampling design and sample size

The sample was selected in two stages. Firstly, the villages served as the Primary Sampling Units (PSUs) were selected by probability proportional to size (PPS), followed by systematic random selection of eligible respondents within each selected PSUs in the second stage. The stratification at the PSU level is carried out by taking the number of households in each PSU as an explicit variable and percentage of SC/ST population and female literacy rate as implicit variables. The list of villages available in Census, 2011 served as a sampling frame for the selection of PSUs. In every selected PSU in intervention and control areas, a mapping and household listing operation was done. This activity identified households with at least one adolescent girl or pregnant woman or mother of a child under two years. From this list, the eligible respondents were selected. A ‘village’ was considered as a unit of at least 500 households. Therefore, small villages (with less than 500 households) were merged with the adjacent village to fulfil the criteria of at least 500 households. Following this, these villages were segmented at least into three segments, and two segments were selected using the systematic PPS method. The household listing was done in the selected PSUs segments only.

A representative sample of 6250 adolescent girls (10–19 years), 2573 pregnant women (15–49 years) and 8755 mothers of children under age two years (15–49 years) and their children (<2 years) were interviewed from five blocks of Bihar, Chhattisgarh, and Odisha at baseline. While for the endline survey, a representative sample of 4241 adolescent girls (10–19 years), 1698 pregnant women (15–49 years) and 4766 mothers of children under age two years (15–49 years) were interviewed from five blocks of Bihar, Chhattisgarh, and Odisha. The sample size accounted for a 10% non-response rate and 1.5 design effect. The details of the sample size are given in S2 Fig. For the purpose of the analysis, the baseline and endline data for the three states was clubbed.

### 2.6 Survey tools

For the survey, standardised, bilingual questionnaires (English and local language) were administered to participants by 24 trained field investigators in each state and anthropometric (weight, height and MUAC) measurements were taken. Data were collected using Census and Survey Processing System (CS Pro) and analysed using STATA 15.

### 2.7 Ethical clearance

The endline survey protocol, methodology and tools were approved by the Institutional Ethics Committee of the International Institute of Population Sciences, Mumbai on 16^th^ February 2021 and conformed to answer the questions and commitments made by the study as registered with the Registry for International Development Impact Evaluations (RIDIE-STUDY-ID-58261b2f46876) and Indian Council of Medical Research (ICMR) National Clinical Trials Registry of India (CTRI/2016/11/007482) [17]. Written consent was taken from all the participants before conducting the interviews.

### 2.8 Study outcomes

BMI was used to assess the nutrition status of adolescent girls and mother of children under 2 years. BMI is a common anthropometric measure which is calculated by dividing a person’s weight in kilograms by the square of their height in meters. The resulting number is used to categorize individuals into different BMI ranges, such as underweight, normal weight, overweight, and obesity. The standard cut-off of <18.5 kg/m^2^ was used to identify underweight [25]. BMI is generally not used for pregnant women because pregnancy brings about significant physiological changes in a woman’s body and might not accurately reflect the nutritional status of pregnant women [26].

Therefore, Mid-Upper Arm Circumference (MUAC) with cut-offs of <23 cm for any malnutrition is being increasingly used for pregnant women. The MUAC measurement involves using a flexible measuring tape to determine the circumference of the upper arm at the midpoint between the shoulder and elbow. It provides an indication of muscle mass and fat stores in the upper body, which can be useful for identifying acute malnutrition and nutritional deficiencies.

The trends and patterns in outcomes related to studying the 1) extent of decline in proportion of adolescents and mothers with children under two with low BMI 2) increment of 0.4 cm in mean MUAC among pregnant women and 3) coverage of 18 key nutrition-specific and sensitive interventions which have been well recognized to address immediate and underlying causes of undernutrition [27–29] (mentioned in S2 Table). In addition, the percentage of women who were identified as nutritionally at-risk was also analysed.

### 2.9 Explanatory variables

The independent variables used in the analyses included girl and women’s age and education, household’s social class and religion and wealth quintile. The age of pregnant women and mothers of children under 2 years was coded in three categories viz. a) less than 20 years, b) 20 to 29 years and c) more than 30 years. Two categories of age were created for adolescent girls: 10–14 years and 15 to 19 years.

Education was categorized as no education, primary, secondary; and higher secondary and above. For adolescent girls’ education was categorized as not attending school and currently attending school. The categories of caste were based on the administrative classification adopted by Government of India and include: Schedule Caste (SC), Schedule Tribe (ST), Other Backward caste (OBC) and Others. Religion was categorized as Hindu and Others which included Muslim and other religious groups such as Sikh and Christian. Wealth quintile was also created based on the number of household assets.

For pregnant women and mother of children under 2 years. the key independent variable of interest was the participation status in participatory learning and action meeting (maitri baithaks) and monthly fixed health camps (VHSND). For the adolescent girls, the key independent variables were participation in AHD and participatory learning and action meeting (Kishori baithaks). In addition, the frequency of participation in the PLA meetings as well as AHDs and VHSNDs over the last three years was studied. The categories of frequency of participation were analysed: no participation, 1–5 times; and at least 6 times.

### 2.10 Statistical analysis

The differential effects were tested by using regression models that estimated changes over time between the 2 areas: intervention and Control area. The rationale behind the difference-in-difference (DID) approach was that the two groups were expected to trend consistently over time. Here the counterfactual was that the implementation of the programme in intervention area should be associated with improvement in utilization of nutrition services and outcomes. The effects were estimated using the following model where, Y denotes the outcome related variables. D_1_ takes value one if it is endline and 0 if baseline. D_2_ takes value 1 for intervention area and zero otherwise.


$$Y_{t}=\beta_{1}^{}+{\beta_{2}^{}D_{1}+\beta_{3}^{}D_{2}+\beta_{2}^{}D_{1}D_{2}+\mu }_{t}^{}$$

To elaborate, the product of D_1_ *and* D_2_ referred to the impact of programme in intervention area organized through the SHGs.

To assess the impact of participation, the groups were defined based on the participation in VHSND and PLA meetings organized by Poshan Sakhis. Categories for the groups were defined for Intervention as well as Control area as: a) participated in VHSND and PLA meetings in Intervention area; participated in VHSND in Control area and b) did not participate in Intervention or Control area.

To establish the association between improvement in service uptake and participation, a logistic regression model was estimated in which the dependent variable considered two values one in case the respondent availed the services and zero otherwise.

$${logit\;\pi }_{i}=\beta_{1}^{}+\beta_{2}^{}{X_{i}^{}}_{}^{}$$

where β_1_ = β_0_ + μ_l_

π_i_ = Pr (y_i_ = 1). Y_i_ is binary and assumes a value 1 if the respondent availed service from the nutrition package. *X*_i_ here refers to the participation variables and other soci-economic correlates. Participation variable is binary in nature with 1 representing participation in programme activities and 0 otherwise. β_0_ is the log of odds of availing the service for reference group for all the categorical variables. Similar models were estimated for nutrition outcome and services with a better outcome being designated value 1 and 0 otherwise. Similarly, full adjusted models were estimated with frequency of participation as the independent variable.

## 3. Results

The socio-economics characteristics of adolescent girls, pregnant women and mothers of children under two years in intervention and control areas are presented in Table 1. In the intervention area, 51.3% of adolescent girls were aged 15–19 years while 66.1% of pregnant women and 69.7% of mothers of children under two years were 20–29 years of age. In control area, 51% of adolescent girls were aged 15–19 years while 69.6% of pregnant women and 68.9% of mothers of children under two years were 20–29 years of age.

**Table 1:** Socio-economic characteristics across intervention and control area, endline survey, 2021.

	Adolescent Girls			Pregnant woman			Mother of children under 2 years		
	Control	Intervention		Control	Intervention		Control	Intervention	
N	2104	2137	P value	882	816	P value	2510	2256	P value
Age			0.548			0.01			0.526
10–14 years	49	48.7		-	-		-	-	
15–19 years	51	51.3		12.4	14.3		6.8	7.9	
20–29 years	-	-		69.6	66.1		68.9	69.7	
30 and above	-	-		17.9	19.5		24.3	22.4	
Education			0.367						
Currently attending school									
No	34.2	33		-	-		-	-	
Yes	65.8	67		-	-		-	-	
Educational attainment						0.171			0.211
No education	-	-		38.2	35		41.3	37.9	
Primary	-	-		27.7	32.3		31.2	31.4	
Secondary	-	-		19.4	17.2		16.5	18.1	
Higher secondary and above	-	-		14.7	15.5		11	12.6	
Religion			0			0.769			0.002
Others	26.5	17.9		34.2	25		31.3	24.4	
Hindu	73.5	82.1		65.8	75		68.7	75.6	
Caste			0.114			0.242			0.153
SC	28.1	27.3		22.3	26.7		22.5	23.6	
ST	31	37.3		30.2	36.4		34.4	37.4	
OBC/Other	40.8	35.4		47.5	36.9		43.1	39	
Wealth			0.916			0.954			0.956
Bottom 50	50.7	49.4		51.8	51		51.6	50.2	
Top 50	49.3	50.6		48.2	49		48.4	49.8	
Total	100	100		100	100		100	100	

Note: All p-values are produced using Chi-square test and indicate that the distribution does not vary across control and intervention area.

One in three adolescent girls were not attending school in control and intervention area. More than 30% of pregnant women and mothers of children under 2 had no education across both control and intervention areas. More than 70% of adolescent girls and women were Hindu and one-fourth were from the Schedule Caste category.

The frequency of participation in programme activities in intervention and control area over the last 3 years was estimated (Table 2). We defined participation on the basis of frequency of contacts: 0 contacts, 1–5 contacts and 6 and more contacts. Among adolescent girls, approximately 9% attended AHD and 11% attended PLA meetings atleast 6 times during the last 3 years in the intervention area. Approximately 8% pregnant woman and 12% mother of children under 2 years participated in PLA meetings at least 6 times during the last 3 years in the intervention area. More than 23% mothers of children under 2 attended VHSND at least 6 times. Participation of adolescent girls attending PLA meetings was low: 73% did not attend any PLA meetings during the last 3 years.

**Table 2:** Frequency of participation in Swabhimaan programme activities (PLA meeting, VHSND and AHD) across intervention and ontrol area, endline survey, 2021.

	Adolescent girls		Pregnant woman		Mother of children under 2 years		Adolescent girls	Pregnant woman	Mother of children under 2 years
	Attended AHD	Attended PLA Meeting	Attended VHSND	Attended PLA Meeting	Attended VHSND	Attended PLA Meeting	Attended AHD	Attended VHSND	Attended VHSND
N	2137	2137	816	816	2256	2256	2104	882	2510
0 contacts	61.9	73	46.9	67.3	40.3	62.9	85.3	57.7	54
1 contact	10	3.3	12.2	6.6	8.7	4.2	4.4	9.4	6.7
2 contacts	8.2	3.9	9.4	8	8.8	8	3.5	8.5	7
3 contacts	4.8	3.6	7.3	4.2	7.3	5	2.1	6.8	5.2
4 contacts	3.5	2.9	5.3	3.9	5.6	4	0.9	4.5	4.5
5 contacts	2.3	2.2	4.1	2.1	5.4	3.7	1.2	3	4.6
6+ contacts	9.1	11	14.9	8	23.9	12.3	2.5	10.1	18
Total	100	100	100	100	100	100	100	100	100

Note: Figures correspond to participation (in % terms) during 3 years in AHD(VHSND) and PLA meetings

At the endline, participation in AHD and VHSND was observed to be higher in the intervention area as compared to the control area (Table 3). A higher percentage of mothers of children under 2 years (33.4%) attended AHD or VHSNDs and PLA meetings. While 28% of pregnant women and 19% adolescent girls attended both in the intervention area, participation of adolescent girls in AHD was low in the control area (14.7%).

**Table 3:** Participation status(percentage) in Swabhimaan programme activities (PLA meeting, VHSND and AHD) across intervention and control areas, endline survey, 2021.

	Adolescent Girls		Pregnant woman		Mother of children under 2 years	
	Intervention	Control	Intervention	Control	Intervention	Control
N	2137	2104	816	882	2256	2510
Attended AHD (VHSND)	38.1	14.7	53.1	42.3	59.7	46
Attended Baithak	27	NA	32.7	NA	37.1	NA
Attended AHD(VHSND) and Baithak	19	NA	28	NA	33.4	NA
No participation	51.9	85.3	38.6	57.7	33.7	54
Any participation	48.1	14.7	61.4	42.3	66.3	46

Note: Participation for intervention area is defined as participation in baithak and VHSND(AHD) during last 3 years while for Control area is defined as participation in VHSND(AHD) during last 3 years.

State-wise analysis reveals that at the endline, participation in AHD and VHSND was observed to be higher in the intervention area as compared to the control area across all the selected blocks (S3 Table). Among pregnant women, 32.7% in Chhattisgarh, 26% in Bihar and 23.6% in Odisha attended AHD/ VHSNDs and PLA meetings. While among mothers of children under 2 years, 34.8% in Odisha, 34.2% in Chhattisgarh and 31.8% in Bihar attended AHD/ VHSNDs and PLA meetings. The participation of adolescents in AHD and PLA meeting was 23.9% in Chhattisgarh, 16.9% in Bihar and 16.3% in Odisha.

In the intervention area, of the 2137 adolescent girls, 13.4% were identified at nutrition risk of whom 8.6% received home visits (Table 4). Among pregnant woman approximately 18% were identified at risk, of whom 13% received a home visit and 11.5% of mothers of children under 2 years received a home visit. Table 2 also presents the receipt of home visit, kitchen garden and food demonstration for only those who were identified at risk. Among those identified at risk more than 60% adolescent girls received home visit while more than 70% of pregnant women and mother of children under 2 years received home visit. It is noted that most of the women who were identified at-risk received home visits and were engaged in food demonstration activities aimed at improving food habits and dietary diversity.

**Table 4:** Nutritionally at-risk and received home visits, have a kitchen garden, and received food demonstration in intervention area, endline survey, 2021.

	Adolescent Girls N(%)	Pregnant woman N(%)	Mother of children under 2 years N(%)
	2137 (100)	816 (100)	2256 (100)
Identified at risk and received home visit	184 (8.6)	106 (13)	259 (11.5)
Identified at risk and did not receive home visit	103 (4.8)	43 (5.3)	95 (4.2)
Women at nutrition risk	287(100)	149(100)	354(100)
received home visit	184(64.2)	106(71)	259(73.2)
attended food demonstration and counseling session	138(48.1)	87(58.1)	97(27.4)
household have a kitchen garden	171(59.7)	82(55.2)	212(59.9)
received home visit, attended food demonstration, and have a kitchen garden	63(22.2)	44(29.3)	48(13.6)
received and either received home visit, attended food demonstration, or have a kitchen garden	251(87.7)	134(89.7)	322(90.9)

Coverage of nutrition sensitive intervention package for adolescent girls aged 10–19 years was estimated for control and intervention area at both baseline and endline (Table 5). Minimum dietary diversity improved from 17.8% at baseline to 38% at endline in the intervention area. Nutritional indictors improved substantially for those who have attended both AHD and PLA meeting as compared to those who did not. A higher percentage of participants reported having kitchen gardens (71.8%) as compared to non-participants (52.4%) at endline in the intervention area. Among adolescents participating in both PLA meeting and VHSNDs, approximately 80% reported use of sanitary pads in intervention area. The percentage of adolescent girls consuming 4 or more IFA tablets was 22.4% at the endline in the intervention area compared to baseline (8.9%). Nutritional status of those adolescent girls who were exposed to programme activities (BMI<18.5) was better at endline (10.5%) compared to baseline (17.5%).

**Table 5:** Access to nutrition specific and nutrition sensitive intervention package for adolescent girls age 10–19 years across intervention and control area at baseline (2016) and endline (2021) by participation status in AHD and PLA meeting.

	Intervention		Control			Intervention		Control	
	Baseline 2016	Endline 2021	Baseline 2016	Endline 2021	DID	Endline 2021	Endline 2021	Endline 2021	Endline 2021
	Overall	Overall	Overall	Overall		Attended AHD+ PLA meeting	No participation	Attended AHD	No participation
N	2986	2137	3264	2104		406	1109	309	1795
Improve food and nutrient intake									
Minimum dietary diversity (6 out of 10 food groups) (%)	17.8	38.8	17.5	36.8	1.67	38	36.9	43.7	35.7
Living in a household with iodised salt (%)	79.6	98.1	76.4	97.7	-2.71*	97.7	97.7	99.1	97.4
Living in households with a kitchen garden (%)	43.2	52.4	35.7	45.6	-0.66	71.8	42.1	61.5	42.8
Increase access to education sanitation and commodities for WASH									
Living in households which do not practice open defecation (%)	18.3	58.7	17.6	59.6	-1.58	70.4	53.9	54.2	60.5
Using safe pads or sanitary pads (%)	37.9	72.5	38	66.5	6.01**	80.2	65.1	82	63.6
Prevent micronutrient deficiencies and anaemia									
Consumed 4 or more IFA tablets (%)	8.9	17.6	11.8	17.6	2.81	22.4	11.1	33	15
Consumed deworming tablets (%)	47.9	75.3	48.4	70.3	4.12	78.5	72.5	70.4	70.3
Increase access to health services and special care to nutritionally ‘at-risk’ adolescent (BMI<18.25)									
Adolescent girls who visit Anganwadi Centre (AWC) for any service (%)	22.2	41.5	28.5	32.4	15.43***	68.7	24.2	60	27.7
Nutritional Status									
Thin (BMI<18.5) (%)	17.5	13	15.1	12.2	-1.66	10.5	14.8	10.3	12.6
Experiencing double burden of short and thin (%)	8.2	3.7	6.2	3.6	-1.90*	2.8	4.1	1.7	3.9

Note: Difference-in-difference (DID) here refers to change in outcome over time between intervention and Control area. Level of significance

*P < 0.10

**P < 0.05

***P < 0.01.

Pregnant women at the endline in intervention areas as compared to control area reported higher improvement in coverage of ICDS entitlement, consumption of calcium tablets and access to health care services (Table 6). In fact, those pregnant women in the intervention areas who participated in both the VHND and PLA meetings reported higher consumption of calcium as compared to non-participants (77.2 vs 50.7) and received first antenatal care in the first trimester (81.4 vs 57.3).

**Table 6:** Access to nutrition specific and nutrition sensitive intervention package among pregnant women, Swabhimaan programme across intervention and control area at baseline (2016) and endline (2021) by participation status in VHSND and PLA meeting.

	Intervention		Control			Intervention		Control	
	Baseline 2016	Endline 2021	Baseline 2016	Endline 2021	DID	Endline 2021	Endline 2021	Endline 2021	Endline 2021
	Overall	Overall	Overall	Overall		Attended VHSND+ PLA meeting	No participation	Attended VHSND	No participation
N	1277	816	1296	882		228	315	373	509
Improve food and nutrient intake									
Minimum dietary diversity (6 out of 10 food groups) (%)	20.6	44.3	21.4	38.9	6.28	56.7	34.9	48.9	31.7
Living in a household with iodized salt (%)	79.4	98.8	73.5	96.8	-3.98*	98.9	98.9	96.9	96.8
Living in food secure households (%)	25.2	35.2	21.8	34.9	-3.11	39	37.9	35.6	34.4
Living in households with a kitchen garden (%)	35.9	50.3	31.1	39.3	6.19	69.2	33.7	52.5	29.6
Received ICDS entitlement for supplementary food in month preceding survey (%)	27.8	65	32.6	55.2	14.62***	69.1	57.4	58.2	53.1
Increase access to education and commodities for WASH									
Living in households which do not practice open defecation (%)	16.4	56.7	13	59	-5.75	64.9	53	58.9	59
Prevent micronutrient deficiencies and anaemia									
Consumed 25 or more IFA tablets in second third trimester (%)	23.6	64.4	24.8	62.7	2.89	66.6	58	67.8	58.7
Two calcium tablets in second trimester (%)	17	62.1	22.8	54.4	13.46***	77.2	50.7	59.6	50.5
Prevent early, poorly spaced or unwanted pregnancies									
Using a modern family planning method before current pregnancy (%)	6.7	16.7	6.9	12.7	1.83	22.3	8.7	18.4	8.4
Decisions about their own health care (%)	72.5	73.7	71.4	69.9	2.77	64.2	80.8	68.9	70.6
Decisions about making major purchases for the household (%)	71.3	73.3	72.7	69.9	4.77	64.4	82.1	69.2	70.4
Taking decisions about visits to family members or relatives (%)	65.8	71.9	66.4	68	4.52	63.5	81	67.6	68.3
Increase access to health services and special care to nutritionally ‘at-risk’ women (MUAC <23cm)									
First antenatal checkup in first trimester (%)	41.1	67.5	47.8	58.1	16.14***	81.4	57.3	69.7	49.6
Weighed at least once in first trimester (%)	72.9	85.8	78.5	76.2	15.18***	92.7	79.4	79.8	73.7
Height was recorded (%)	10.4	31.5	11.2	21.4	11.00***	47.8	18.6	22.1	20.8
Nutritional Status (%)									
MUAC<23 cm	41	37.1	45.1	34.5	6.59	34.7	36.3	31.7	36.6

Note: Difference-in-difference (DID) here refers to change in outcome over time between intervention and Control area. Level of significance

*P < 0.10

**P < 0.05

***P < 0.01.

Improvements were noticed in consumption of diversified diet, IFA tablets and access to healthcare services among those mothers with children under 2 years who had attended VHSND and PLA meetings as compared to those who did not participate (Table 7). In mothers with children under 2 years, a substantial difference was observed in the consumption of diversified diet (49 vs 36.2%), IFA tablets (48.2 vs 31.7%), use of modern family planning methods (40.5 vs 22.5%), and receipt of four ANC services (51.5 vs 36.7%).

**Table 7:** Access to nutrition specific and nutrition sensitive intervention package among mothers with children under 2 years of age across intervention and control areas at baseline (2016) and endline (2021) by participation status in VHSND and PLA meeting.

	Intervention		Control			Intervention		Control	
	Baseline 2016	Endline 2021	Baseline 2016	Endline 2021	(DID)	Endline 2021	Endline 2021	Endline 2021	Endline 2021
	Overall	Overall	Overall	Overall		Attended VHSND+ PLA meeting	No participation	Attended VHSND	No participation
N	4441	2256	4314	2510		754	760	1155	1355
Improve food and nutrient intake									
Minimum dietary diversity (6 out of 10 food groups) (%)	19.4	44.1	18.2	40.2	2.79	49	36.2	46	35.1
Living in a household with iodized salt (%)	75.7	98.4	74.4	97.2	-0.08	99.2	97.4	96.7	97.6
Living in food secure households (%)	22.1	29.4	21.7	29.1	-0.20	30.3	30.9	28.4	29.8
Living in households with a kitchen garden (%)	32.5	51.6	29.9	38.3	10.63***	66.5	37.6	45.2	32.5
Received minimum PDS entitlement in month preceding survey (%)	70.3	93.8	73.7	93.2	3.97**	95.4	94.7	91.8	94.5
Received ICDS entitlement for supplementary food in month preceding survey (%)	44.5	78.6	49.7	76.4	7.38***	84.6	72.6	77.2	75.7
Increase access to education and commodities for WASH									
Living in households which do not practice open defecation (%)	19.7	58.7	14.3	56.5	-3.21	62.3	59.6	54.6	58.1
Prevent micronutrient deficiencies and anaemia									
Consumed 100 or more IFA tablets during last pregnancy (%)	22.4	38.8	32.7	38.6	10.52***	48.2	31.7	45.4	32.8
Consumed 100 or more calcium tablets last pregnancy (%)	0.1	28.7	0.1	26.5	2.16	34.7	24.7	32.2	21.6
Prevent early, poorly spaced or unwanted pregnancies									
Using a modern family planning method (%)	13	30.5	13.5	23	8.01***	40.5	22.5	25	21.2
Taking decisions about their own health care (%)	67.1	72.1	69.9	74.6	0.31	69.4	73.4	73.5	75.5
Taking decisions about making major purchases for the household (%)	68.7	71.9	71.9	73.2	1.89	68.5	73.7	71.9	74.3
Taking decisions about visits to family members/relatives (%)	65.3	71.4	69.7	74.1	1.66	69.7	73.2	72.1	75.8
Increase access to health services and special care to nutritionally ‘at-risk’ women (MUAC <23cm)									
First antenatal checkup in first trimester (%)	30.1	64.9	25.9	58.5	2.11	70.2	56.8	64	53.8
Received at least 4 antenatal care in last pregnancy (%)	18.2	43.3	20.8	39	6.84***	51.5	36.7	43.1	35.5
Height was recorded (%)	14.9	38.6	13.4	26.9	10.25***	51.4	28.5	27.6	26.4
Weighed at least four times in last pregnancy (%)	22.3	36.3	24.7	31	7.67***	41.3	28.9	37.7	25.4
Accessed JSY	51.7	53.8	53.3	48	7.33***	59.2	49	49.5	46.7
Delivered in a health facility in last pregnancy (%)	73.3	83.7	69.1	75.6	3.83*	81.5	83.3	77.8	73.8
Nutritional Status									
Mothers who are thin (BMI<18.5) (%)	49	38.3	49.9	38.6	0.66	37.9	38.7	39	38.3

Note: Difference-in-difference (DID) here refers to change in outcome over time between intervention and Control area. Level of significance

*P < 0.10

**P < 0.05

***P < 0.01.

Multivariable logistic regression was applied to endline data to examine the association between receipt of nutrition specific services and nutrition outcomes with status of participation adjusted for socio-economic characteristics (Table 8). Here the participation status is defined based on participation in both AHD or VHSND and PLA meetings. Results indicated that participation status was associated with higher odds of consumption of micronutrients across all the three groups comprising adolescent girls, pregnant women and mothers of under two. For instance, consumption of IFA was 47% higher among adolescent girls [OR = 1.47, 95% CI = 1.07–2.02] and 78% among mothers [OR = 1.78, 95% CI = 1.42–2.22] who participated in meetings. Similarly, the odds of consumption of calcium were two times higher among pregnant women [OR = 2.60, 95% CI = 1.76–3.83] and more than 50% among mothers [OR = 1.52, 95% CI = 1.21–1.93] who participated in meetings. The odds of utilization of health services were higher among pregnant woman and mother of children under 2 years who participated in meetings. In particular, for pregnant women, there was 2-fold higher odds of check-up in first trimester [OR = 2.55, 95% CI = 1.68–3.88] and receipt of ANC visits [OR = 2.58, 95% CI = 1.82–3.64] with increased participation in the meetings. Apart from utilization of services, improvement in intake of diversified diet among pregnant and mothers was observed The odds of using sanitary pads were 50% higher among participants.

**Table 8:** Multivariable logistic regression of association between receipt of nutrition specific services and nutrition outcomes with participation status adjusted for socio-economic characteristics, endline survey, 2021.

	Adolescent Girls				Pregnant Women				Mother of children under 2 years			
	Odds ratio	CI	N	pseudo R-sq	Odds ratio	CI	N	pseudo R-sq	Odds ratio	CI	N	pseudo R-sq
Improve food and nutrient intake												
Minimum dietary diversity (6 out of 10 food groups)	0.95	(0.71–1.28)	1749	0.039	2.05***	(1.41–2.99)	675	0.056	1.38*	(1.08–1.77)	1758	0.046
Living in a household with iodized salt	0.64	(0.25–1.66)	2135	0.043	1.16	(0.19–7.18)	668	0.119	2.39	(1.00–5.73)	2255	0.031
Living in food secure households		-			1.23	(0.87–1.73)	811	0.089	0.98	(0.78–1.24)	2252	0.085
Living in households with a kitchen garden	2.68***	(2.05–3.50)	2137	0.044	3.07***	(2.16–4.37)	815	0.098	2.34***	(1.87–2.94)	2256	0.074
Received minimum PDS entitlement in month preceding survey		-	-	-		-	-	-	1.36	(0.87–2.13)	1932	0.065
Received ICDS entitlement for supplementary food in month preceding survey #	4.09***	(3.15–5.32)	2137	0.064	1.08	(0.74–1.57)	815	0.139	1.51*	(1.07–2.13)	2256	0.199
Increase access to education sanitation and commodities for WASH												
Living in households which do not practice open defecation	1.80***	(1.38–2.36)	2137	0.078	1.59**	(1.13–2.26)	815	0.116	1.14	(0.91–1.42)	2256	0.087
Percentage of using safe pads or sanitary pads	1.64**	(1.20–2.22)	1822	0.077		-				-		
Consumed IFA tablets##	1.47*	(1.07–2.02)	2137	0.015	1.12	(0.76–1.64)	658	0.019	1.78***	(1.42–2.22)	2104	0.038
Consumed calcium tablets ###		-			2.60***	(1.76–3.83)	772	0.053	1.52***	(1.21–1.93)	2256	0.020
Consumed deworming tablets (%)	1.89***	(1.41–2.54)	1978	0.037		-				-		
Prevent early, poorly spaced or unwanted pregnancies												
Using a modern family planning method	-	-	-	-	1.70*	(1.11–2.60)	815	0.033	1.83***	(1.47–2.29)	2256	0.039
Taking decisions about their own health care	-	-	-	-	0.53***	(0.37–0.75)	814	0.021	0.83	(0.66–1.05)	2256	0.011
Taking decisions about making major purchases for the household	-	-	-	-	0.55***	(0.39–0.78)	814	0.021	0.79*	(0.63–1.00)	2256	0.009
Taking decisions about visits to family members or relatives	-	-	-	-	0.58**	(0.41–0.82)	814	0.016	0.88	(0.70–1.10)	2256	0.006
Increase access to health services												
First antenatal checkup in first trimester	-	-	-	-	2.55***	(1.68–3.88)	815	0.080	1.36*	(1.07–1.73)	2256	0.038
Received antenatal care	-	-	-	-		-			1.62***	(1.30–2.02)	2256	0.052
Height was recorded	-	-	-	-	2.58***	(1.82–3.64)	815	0.078	2.06***	(1.67–2.55)	2256	0.048
Weighed ####	-	-	-	-	2.29**	(1.23–4.25)	815	0.106	1.31*	(1.04–1.64)	2256	0.047
Accessed JSY	-	-	-	-		-	-	-	1.44***	(1.16–1.77)	2256	0.016
Delivered in a health facility in last pregnancy	-	-	-	-		-	-	-	0.82	(0.64–1.05)	2256	0.041
Nutritional Status												
Thin	0.81	(0.55–1.18)	2122	0.027	0.93	(0.65–1.32)	815	0.047	1.00	(0.81–1.24)	2251	0.013

#Received ICDS in month preceding the survey for Mothers, use of AWC services for adolescent girls

##IFA consumption is 4 or more for adolescent girls, 25 or more for pregnant woman and 100 or more for mothers

### Calcium tablets for pregnant woman and 100 or more for mothers

#### weighed refers to atleast 1 for pregnant woman and four times for mothers

@thin is defined as BMI <18.5 for adolescent girls and mothers, MUAC less than 23 cm for pregnant woman.

The results of multivariable logistic regression model which examines the association between receipt of nutrition specific services and nutrition outcomes with frequency of participation status in group meetings and AHDs and VHSNDs respectively, adjusted for socio-economic characteristics were also estimated (Table 9 and S10 Table).

**Table 9:** Multivariable logistic regression of association between receipt of nutrition specific services and nutrition outcomes with frequency of participation in PLA meeting adjusted for socio-economic characteristics, endline survey, 2021.

	Adolescent Girls		Pregnant women		Mother of children under 2 years	
	1 to 5 contacts	6+ contacts	1 to 5 contacts	6+ contacts	1 to 5 contacts	6+ contacts
Improve food and nutrient intake						
Minimum dietary diversity (6 out of 10 food groups)	1.04	1.17	1.74**	1.85*	1.60***	1.69***
Living in a household with iodised salt	1.15	0.5	0.5	0.44	1.78	2.16
Living in food secure households			0.92	1.03	0.86	0.92
Living in households with a kitchen garden	2.05***	2.70***	3.04***	3.59***	1.56***	2.83***
Received minimum PDS entitlement in month preceding survey					1.02	0.54*
Received ICDS entitlement for supplementary food in month preceding survey	4.29***	3.76***	1.83**	1.16	1.70**	1.39
Increase access to education sanitation and commodities for WASH						
Living in households which do not practice open defecation	1.35*	2.10***	1.75**	1.02	1.02	0.76*
Percentage of using safe pads or sanitary pads	1.66***	2.43***				
Prevent micronutrient deficiencies and anaemia						
Consumed IFA tablets	1.61**	2.14**	1.26	2.09*	1.03	3.05***
Consumed calcium tablets			1.92***	3.56***	0.86	2.57***
Consumed deworming tablets (%)	1.70***	1.59*				
Prevent early, poorly spaced or unwanted pregnancies						
Using a modern family planning method			1.69*	2.99***	1.17	2.11***
Taking decisions about their own health care			0.51***	0.63	0.73**	1.31
Taking decisions about making major purchases for the household			0.51***	0.69	0.72**	1.35
Taking decisions about visits to family members or relatives			0.49***	0.59*	0.75*	1.11
Increase access to health services						
First antenatal checkup in first trimester			2.00***	3.22***	1.49**	1.91***
Received antenatal care					0.83	2.74***
Height was recorded			2.61***	1.33	1.91***	0.92
Weighed			3.84***	1.48	0.77*	3.39***
Accessed JSY					1.03	1.91***
Delivered in a health facility in last pregnancy					0.85	1.52*
Nutritional Status						
Thin	0.84	0.51*	0.92	1.57*	1.02	0.79

Note: Reference category is 0 contacts for each target group. Adjusted for socio-economic characteristics of the respondent such as age, education, wealth, social group and religion. Level of significance

*P < 0.10

**P < 0.05

***P < 0.01. Figures in parenthesis are 95% confidence interval.

For adolescents, those who participated in at least 6 AHD or 6 PLA meetings had 2 times higher odds of having a kitchen garden. Moreover, those adolescents who attended more than 6 PLA meeting were twice as likely to use sanitary pads, consume IFA tablets, deworming tablets and visit rural care centre (Anganwadi centre (AWC)) for availing ICDS services. Similarly, model estimated for pregnant women indicated that those who participated more than 6 times in VHSNDs were twice as likely to consume a diversified diet, had 3 times higher odds of living in the household with kitchen and twice more likely to consume micronutrients and use family planning methods. Likewise, mothers who participated were more than 6 times in VHSNDs were likely to receive 4 or more ANC services and consume 100 or more IFA and calcium tablets. The odds of heigh and weight being measured were higher (OR = 2.61 and 3.84 respectively) among pregnant woman who had attended 1 to 5 meetings. and mother of children under 2 years who participated in meetings. Mothers who had attended 1to 5 meetings were 2 times more likely to report that height was measured while the odds of being weighed were four times among those mothers who had participated more than 6 times in meetings.

Coverage of nutrition sensitive intervention package for adolescent girls, pregnant women and mothers of children under 2 years was estimated for control and intervention area across selected blocks at both baseline and endline (S4–S6 Tables). Minimum dietary diversity among adolescents improved from 12.3% at baseline to 50% at endline in the intervention area in Bihar. A higher percentage of adolescents reported use of sanitary pads at endline in both intervention and control areas in all the States. Use of sanitary pads among adolescents improved from 30.3% at baseline to 74.7% at endline in the intervention area in Bihar.

Significant improvement in coverage of ICDS entitlement (DID:18.5pp), consumption of calcium tablets (DID:18.4 pp) and first antenatal check-up in first trimester (DID:19.5pp) was observed among pregnant women at the endline in intervention areas as compared to control area in Bihar (S5 Table). The percentage of pregnant women with MUAC<23 cm decline from 50.2% to 48.9% in intervention area and from 57.3% to 42.9% in control areas in Bihar. The coverage of first antenatal check-up in first trimester and being weighed at least once in first trimester also improved in Chhattisgarh. Among mothers with children under 2 years, a significant difference was observed in the consumption of diversified diet (DID:18.5pp), IFA tablets (DID:14.8 pp), receipt of four ANC services (DID:11.5 pp), and institutional deliveries (DID:12.3 pp) in intervention areas as compared to control areas in Bihar (S6 Table). Access to health care services improved in Chhattisgarh at endline in both control and intervention areas among mothers of children under 2years.

Coverage of nutrition specific and nutrition sensitive intervention package for adolescent girls 10–19 age groups by frequency of participation were estimated (S7 Table). In the Intervention group. a higher percentage of adolescent girls attended 6 and more contacts AHD or PLA meetings. Those girls with at least 6 times contacts reported a higher consumption of a diversified diet (45.4% vs 38.1%), living in a household with kitchen gardens (70.3% vs 62.9%) and a lower percentage of girls with low BMI or classified thin outcomes (7.7% vs 10.9%) as compared to those who participated less than 6 times. In pregnant women, a significant differential in consumption of micronutrients: consumption of IFA (76.5 vs 59.6%) and calcium (80% vs 67.3%) were observed to be higher among those pregnant women who had more than 6 contacts as compared to those who did not participate (S8 Table). Notably, the consumption of micronutrients such as IFA (58.8 vs 31.15) and calcium (46 vs 24.3%) was observed to be higher among those mothers of under twos who participated in these meetings at least 6 times as compared to those who did not participate at all (S9 Table).

## 4. Discussion

This study examined the impact of a community-based and systems-strengthening interventions via women groups on coverage of nutrition interventions and on nutrition status of women before, during and after pregnancy in five poorest blocks of four districts across three Indian states of Bihar, Odisha, and Chhattisgarh. This is one of the first studies to our knowledge that evaluates the impact of engaging women’s collectives with the support of dedicated trained cadres of community resource persons who are integral part of women collectives for reaching and mobilising adolescent girls and women who are pregnant or mothers of under two.

The study found an improvement in coverage of most of the nutrition service-related indicators in both the Intervention and Control areas. The net improvement in secondary outcomes was in the range of 5–10% for most of the indicators in intervention area at the endline while more than 15% increment in access to health services was noted in the intervention areas at the endline among pregnant and lactating women as compared to the control areas. The role of community resource persons in identifying at risk household with adolescent girls, pregnant women and mothers of under twos through a simple measure of MUAC measure followed by microplanning and targeting efforts towards mobilising and improving their participation in group meetings present evidence that engaging a dedicated cadre selected from within women collectives increases demands as well response by the relevant sectors to improve the coverage of health and nutrition services.

These findings concur with other reports that found a positive impact on utilization of health care services and dietary diversity when Behaviour Change Communication— (BCC) modules were introduced through SHG groups [9, 10, 17–21, 30–35]. It is worth mentioning here that during the last decade evaluation of several behavioural change communication interventions implemented by non-governmental organization were conducted which utilized the PLA based approach to improve maternal and child health outcomes. The findings from EKJUT trials and UPAVAN trials which were conducted in Odisha and Jharkhand indicate that PLA based approach for community workers coupled with home visits can lead to improvement in maternal and child health and nutrition outcomes [22, 30]. The evaluation of Parivartan self-help group project and Jeevika Technical Support Programme in Bihar and UPCMP in Uttar Pradesh reveals that health and nutrition behaviour can be modified by leveraging the SHG platform [31, 32].

On the other hand, the study also found that within the intervention and control areas, the nutrition outcomes service coverage of those who participated in AHD or VHSND as well as PLA meetings were better as compared to those who did not participate at all in any of these or in only PLA. This effect was robust for most of the indicators even after adjusting for a host of socio-economic characteristics. In contrast, a few recent studies have found that the association with exposure did not necessarily lead to improvement in the coverage of services [23, 36]. This association requires therefore more studies to understand whether participation in PLA meetings does have the desired effect on coverage of nutrition services.

The study also revealed that the participation rate in VSHND as well as PLA meeting in the intervention area improved at the endline for pregnant woman and mother of children under 2 years. However, the frequency of participation was low. These findings are in line with a few recent studies that reported participation of women in such group meetings was a challenge [37, 38]. Moreover, it is reported that the participation is context specific and a host of factors such as time, accessibility and socio-economic factors determine participation [37, 38].

The study revealed a significant improvement in nutrition services but the nutritional outcomes were surprisingly similar among participants and non-participants although slightly better for adolescent girls. The latter could be attributed to use of cadre of resource persons specifically trained for working with adolescent girls in one of the states. However, the impact on nutritional status was noted to be minimal. This is agreement with the results of the CARING trial that evaluated the impact of introducing a new cadre of workers in districts of Jharkhand and Odisha with high burden of undernutrition but found no change in nutrition outcomes of children after 18 months of birth but did report improvements in birth outcome and dietary diversity [24]. Similarly, another study which evaluated the impact of delivery of nutrition behaviour change communication intervention through SHGs (Professional Assistance for Development Action (PRADAN) model) in five Indian states also found no impact on anthropometry or diet related indicators of a nutrition behaviour change intervention delivered through SHGs [25]. It is therefore evident that gaining quick improvements in BMI status possibly requires additional approaches/interventions. Although the participatory learning and action approach could augment the knowledge base but the translation of knowledge into increase in utilization of services and resulting in better outcomes is governed to a large extent by socio-economic characteristics and related behaviour patterns which can remain stagnant for a longer period.

The study presents evidence that participation status was associated with higher odds of consumption of micronutrients across all the groups and higher chances of utilization of health services among pregnant woman and mother of children under 2 years. In fact, higher dietary diversity was noted among those pregnant women and mothers of children under 2 years who participated in the community activities. This could be attributable to the focus on developing kitchen gardens as well as food demonstration activities coupled with participatory learning and action meetings. WASH indicators (open defecation status and use of sanitary pads) improved significantly. Improvement in such indicator is well documented to impact on the health and nutritional status of adolescents, women, and children [39, 40].

These findings need to take into consideration some limitations. In particular, the study design involved comparison of outcomes at baseline and endline across control and intervention areas. However, overtime the intervention was scaled up in control areas across certain states such as Chhattisgarh. Such diffusion implies that the substantial improvement in some of the control areas could be due to gradual introduction of programme activities. Also, it is worth noting here that the roll out of the Swabhimaan programme coincided with the launch of the Poshan Abhiyaan in 2018 which aims to improve nutrition amongst children, pregnant women, and lactating mothers. The substantial improvement in coverage of nutrition indicators across both intervention and control area could be due to the strengthening of system actions due to the renewed interest in nutrition interventions due to the Poshan Abhiyaan. However, it was outside the scope of the study to evaluate the impact of Poshan Abhiyaan therefore the results must be interpreted with a little caution.

Another limitation of the study is that the data for the three States was pooled for analysis. Since Bihar is the learning and demonstration site where the programme was completely funded by UNICEF India with financial support for human resources, it is expected that the results for Bihar will be better. Towards this end, the impact of programme for each State was evaluated separately and have been documented for the interested readers [41]. We have presented the major findings based on State-wise analysis in the supplementary section (S3–S6 Tables). Our study results reflect different context in the three States as seen by substantial improvement in coverage of nutrition interventions in Bihar where the Swabhimaan programme was completely funded by UNICEF India with financial support for human resources. On average across all the three States, a higher percentage of mothers of children under 2 years participated in AHD/VHSND and PLA meetings while the participation of adolescents remained low. In intervention areas of Bihar, there was significant improvement in dietary diversity of adolescent girls and mothers. The use of sanitary pads increased significantly among adolescent girls in both intervention and control areas in Bihar. In addition, significant improvement in consumption of micronutrients and access to health services was observed in intervention areas as compared to control areas of Bihar as compared to other two States.

Also, a bias towards ‘best behavior’ response cannot be ruled out since women in the intervention areas during the interviews were aware of the programme and therefore are more likely to respond correctly and positively. On the other hand, the improvement in outcomes related to utilization of services and BMI indicate that there was possibly a positive impact on seeking health services and on influencing dietary behaviors. Interestingly, the frequency of participation was observed to be low. This could be attributed to ‘recall bias’ since it is possibly easier for a person to recall participation in the programmatic activities compared to specifying the frequency of participation in organized meetings. Similar concern is echoed by a recent study which evaluated the effect of nutrition-sensitive agriculture intervention with participatory videos. A discrepancy was found in self-reported coverage of home visits and information obtained from intervention team. The discrepancy was possibly due to a large time gap between the conducting the home visit and endline survey [42].

In addition, although, the respondents were asked about home visits, these questions were addressed to those who were identified as nutritionally at risk. However, due to the COVID-19 pandemic, most of the households were receiving home visits by ICDS frontline and health workers (AWWs/ASHAs or Poshan Sakhi) irrespective of nutritional status, and therefore the frequency of home visits may have been underestimated. Another limitation of the study is the practice of inter-cluster migration of women after marriage that could also affect the results since those who were interviewed at endline were necessarily not those who were exposed to the intervention. Likewise, exposure effect among those who did not participate cannot be ruled out. The add on value of home visits when combined with attending VHSND was therefore not analyzed.

## 5. Conclusion

Several important lessons emerge from this study which could be useful for future women-led poverty alleviation programmes aimed at improving coverage of nutrition services and outcomes of adolescent girls and women. First, community resource persons (Poshan Sakhis and Kishori Sakhis) can play a positive role in mobilizing communities and identifying at-risk households. But the outcome could be context specific which implies that extensive consultations with communities and formative research should be conducted to develop consensus about the hypothesized pathways of impact. Second, it is strongly recommended that the impact evaluation of similar interventions in future should be carried out over extended period to produce more useful information about the sustainability of the desired effects. It should be ensured that beneficiaries receive the intended duration and intensity of exposure to the programme activities before assessment. This will ensure a more accurate and reliable assessment of the program’s effectiveness and impact. In conclusion, the findings reveal that the use of NRLM platform and layering of actions of SHGs with nutrition specific and nutrition sensitive activities is crucial for contributing in improving the nutrition outcomes of women and children. However, there is a need for further research to understand the sustainability, minimum additional cost required and effectiveness of such interventions.

## Supporting information

S1 Fig Evaluation design, Swabhimaan programme. (JPG)

S2 Fig Sample details. (JPG)

S1 Table Year-wise budget contributions for Swabhimaan programme—pilot implementation (2016–21) and scale-up (2018 onwards) (Bihar, Chhattisgarh and Odisha). (DOCX)

S2 Table Essential nutrition interventions of Swabhimaan programme 2016–2021. (DOCX)

S3 Table State-wise participation status in AHD/VHSND and PLA meetings in intervention and control areas, endline survey, 2021. (DOCX)

S4 Table Access to nutrition specific and nutrition sensitive intervention package for adolescent girls age 10–19 years in intervention and control area by participation status in AHD and PLA meeting across states. (DOCX)

S5 Table Access to nutrition specific and nutrition sensitive intervention package among pregnant women, Swabhimaan program in intervention and control area by participation status in VHSND and PLA meeting across states. (DOCX)

S6 Table Access to nutrition specific and nutrition sensitive intervention package among mothers with children under 2 years of age in intervention and control areas by participation status in VHSND and PLA meeting across states. (DOCX)

S7 Table Access to nutrition specific and nutrition sensitive intervention package for adolescent girls age 10–19 years in intervention area by participation status and frequency of participation in AHD and PLA meeting. (DOCX)

S8 Table Access to nutrition specific and nutrition sensitive intervention package among pregnant women in intervention area by participation status and frequency of participation in VHSND and PLA meeting. (DOCX)

S9 Table Access to nutrition specific and nutrition sensitive intervention package among mothers with children under 2 years of age in intervention area by participation status and frequency of participation in VHSND and PLA meeting. (DOCX)

S10 Table Multivariable logistic regression of association between receipt of nutrition specific services and nutrition outcomes with frequency of participation in AHD (VHSND) adjusted for socio-economic characteristics, endline survey, 2021. (DOCX)

S1 Data (ZIP)
