# Supplementary figures and images for: Evaluation of impact of engaging federations of women groups to improve women’s nutrition interventions- before, during and after pregnancy in social and economically backward geographies: Evidence from three eastern Indian States
Source: PLoS One. 2023 Oct 5;18(10):e0291866. doi: 10.1371/journal.pone.0291866 (PMC10553280; doi:10.1371/journal.pone.0291866)

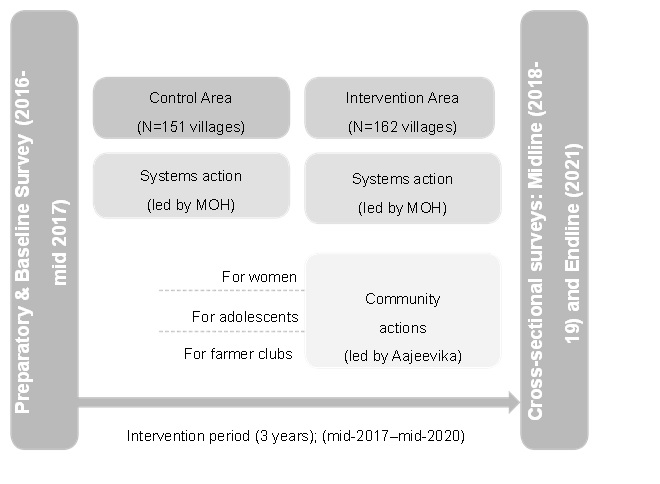

Supplement: S1 Fig — (JPG) [file pone.0291866.s001.jpg]

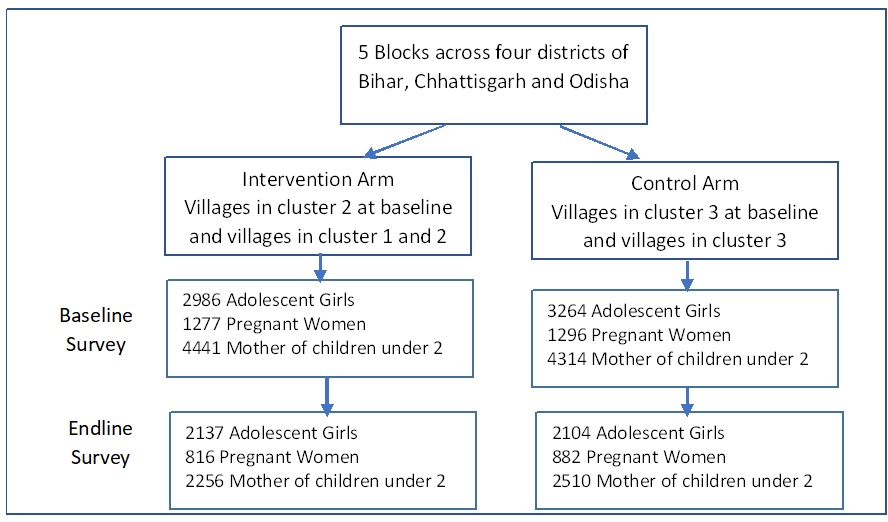

Supplement: S2 Fig — (JPG) [file pone.0291866.s002.jpg]
