# Supplementary material for: Evaluation of impact of engaging federations of women groups to improve women’s nutrition interventions- before, during and after pregnancy in social and economically backward geographies: Evidence from three eastern Indian States
Source: PLoS One. 2023 Oct 5;18(10):e0291866. doi: 10.1371/journal.pone.0291866 (PMC10553280; doi:10.1371/journal.pone.0291866)
Supplement: S1 Table — (DOCX) [file pone.0291866.s003.docx]

**Table S1: Year-wise Budget contributions for Swabhimaan Programme - Pilot Implementation (2016-21) and Scale-up (2018 onwards) (Bihar, Chhattisgarh and Odisha)**

|  |  | **Oct – Dec, 15** | **Jan - Dec, 16** | **Jan - Dec, 17** | **Jan - Dec, 18** | **Jan - Dec, 19** | **Jan - Dec, 20** | **Jan - Dec, 21** | **2015-21 (in INR)** | **2015-21 (in $)** |  |
| --- | --- | --- | --- | --- | --- | --- | --- | --- | --- | --- | --- |
|  |  |  |  |  |  |  |  |  |  |  |  |
| **Bihar** | UNICEF | 22,35,500 | 51,82,565 | 70,53,101 | 41,54,170 | 75,47,533 | 45,33,555 | 20,63,052 | 3,27,69,476 | 4,33,688 |  |
|  | SRLM | - | - | - | - | - | - | - | - |  |  |
|  | **State Total** | 22,35,500 | 51,82,565 | 70,53,101 | 41,54,170 | 75,47,533 | 45,33,555 | 20,63,052 | 3,27,69,476 | 4,33,688 |  |
| **Chhattisgarh** | UNICEF | - | 83,39,000 | 44,64,000 | 65,52,604 | 41,26,216 | 4,23,000 | 13,84,000 | 2,52,88,820 | 3,34,685 |  |
|  | SRLM | - | 84,91,000 | 51,87,000 | 39,11,000 | 2,33,41,000 | 2,22,14,000 | 80,60,200 | 7,12,04,200 | 9,42,353 |  |
|  | **State Total** | - | 1,68,30,000 | 96,51,000 | 1,04,63,604 | 2,74,67,216 | 2,26,37,000 | 94,44,200 | 9,64,93,020 | 12,77,038 |  |
| **Odisha** | UNICEF | - | 84,68,817 | 92,38,807 | 33,31,833 | 79,96,400 | 59,87,433 | 39,83,400 | 3,90,06,690 | 5,16,235 |  |
|  | SRLM | - | 33,52,500 | 90,26,100 | 5,16,51,775 | 4,24,51,425 | 5,90,22,550 | 3,52,50,000 | 20,07,54,350 | 26,56,887 |  |
|  | **State Total** | - | 1,18,21,317 | 1,82,64,907 | 5,49,83,608 | 5,04,47,825 | 6,50,09,983 | 3,92,33,400 | 23,97,61,040 | 31,73,121 |  |
| **National support for NRLM and scale-up for Food, Nutrition, Health and WASH (FNHW)and Gender** | UNICEF | - | - | - | - | 2,20,83,074 | 59,45,425 | 55,12,795 | 3,35,41,294 | 4,43,903 |  |
| **Consolidated budget** | **UNICEF ( INR)** | 22,35,500 | 2,19,90,382 | 2,07,55,908 | 1,40,38,607 | 4,17,53,223 | 1,68,89,413 | 1,29,43,247 | 13,06,06,280 |  |  |
|  | **UNICEF ( USD)** | 29,586 | 2,91,032 | 2,74,694 | 1,85,794 | 5,52,584 | 2,23,523 | 1,71,298 | 17,28,511 | 17,28,511 |  |
|  | **SRLM ( INR)** |  | 1,18,43,500 | 1,42,13,100 | 5,55,62,775 | 6,57,92,425 | 8,12,36,550 | 4,33,10,200 | 27,19,58,550 |  |  |
|  | **SRLM ( USD)** |  | 1,56,743 | 1,88,103 | 7,35,346 | 8,70,731 | 10,75,126 | 5,73,190 | 35,99,240 | 35,99,240 |  |
| **Grand total (in INR)** |  | **22,35,500** | **3,38,33,882** | **3,49,69,008** | **6,96,01,382** | **10,75,45,648** | **9,81,25,963** | **5,62,53,447** | **40,25,64,830** | **-** |  |
| **Grand total (in USD)** |  | **29,586** | **4,47,775** | **4,62,798** | **9,21,141** | **14,23,315** | **12,98,650** | **7,44,487** | **53,27,751** | **53,27,751** |  |

Note: The figures here represent the expenditure incurred towards Swabhimaan programme in the selected blocks: Jalalgarh and Kasba blocks in Purnia district in Bihar, Koraput block in the Koraput district and Pallahara block in the Angul district in Odisha; and Bastar and Bakawand block in Bastar district in Chhattisgarh
