# Supplementary material for: Evaluation of impact of engaging federations of women groups to improve women’s nutrition interventions- before, during and after pregnancy in social and economically backward geographies: Evidence from three eastern Indian States
Source: PLoS One. 2023 Oct 5;18(10):e0291866. doi: 10.1371/journal.pone.0291866 (PMC10553280; doi:10.1371/journal.pone.0291866)
Supplement: S2 Table — (DOCX) [file pone.0291866.s004.docx]

**Table S2. Essential nutrition interventions of Swabhimaan programme 2016-2021.**

|  | Adolescent Girls | Pregnant women | Mother of children under 2 years |
| --- | --- | --- | --- |
| Improve food and nutrient intake |  |  |  |
| Minimum dietary diversity (6 out of 10 food groups) | ✓ | ✓ | ✓ |
| Living in a household with iodized salt | ✓ | ✓ | ✓ |
| Living in food secure households | - | ✓ | ✓ |
| Living in households with a kitchen garden | ✓ | ✓ | ✓ |
| Received minimum PDS entitlement in month preceding survey | - | - | ✓ |
| Received ICDS entitlement for supplementary food in month preceding survey # | ✓ | ✓ | ✓ |
| Increase access to education sanitation and commodities for WASH |  |  |  |
| Living in households which do not practice open defecation | ✓ | ✓ | ✓ |
| Percentage of using safe pads or sanitary pads | ✓ | - | - |
| Consumed IFA tablets## | ✓ | ✓ | ✓ |
| Consumed calcium tablets ### | - | ✓ | ✓ |
| Consumed deworming tablets (%) | ✓ | - | - |
| Prevent early, poorly spaced or unwanted pregnancies and women empowered |  |  |  |
| Using a modern family planning method | - | ✓ | ✓ |
| Taking decisions about their own health care | - | ✓ | ✓ |
| Taking decisions about making major purchases for the household | - | ✓ | ✓ |
| Taking decisions about visits to family members or relatives | - | ✓ | ✓ |
| Increase access to health services |  |  |  |
| First antenatal checkup in first trimester | - | ✓ | ✓ |
| Received antenatal care | - | - | ✓ |
| Height was recorded | - | ✓ | ✓ |
| Weighed #### | - | ✓ | ✓ |
| Accessed JSY | - | - | ✓ |
| Delivered in a health facility in last pregnancy | - | - | ✓ |
| Nutritional Status |  |  |  |
| Thin | ✓ | ✓ | ✓ |

Note: #Received ICDS in month preceding the survey for Mothers while use of AWC services for adolescent girls

##IFA consumption is 4 or more for adolescent girls, 25 or more for pregnant woman and 100 or more for mothers

### Calcium tablets for pregnant woman and 100 or more for mothers

#### weighed refers to atleast 1 measure for pregnant woman and four times for mothers

@thin is defined as BMI <18.5 for adolescent girls and mothers while MUAC less than 23 cm for pregnant woman
