# Supplementary material for: Evaluation of impact of engaging federations of women groups to improve women’s nutrition interventions- before, during and after pregnancy in social and economically backward geographies: Evidence from three eastern Indian States
Source: PLoS One. 2023 Oct 5;18(10):e0291866. doi: 10.1371/journal.pone.0291866 (PMC10553280; doi:10.1371/journal.pone.0291866)
Supplement: S3 Table — (DOCX) [file pone.0291866.s005.docx]

**Table S3: Participation status in AHD/VHSND and PLA meetings in intervention and control areas, Endline survey, 2021**

|  | Adolescent Girls | | Pregnant woman | | Mother of children under 2 years | |
| --- | --- | --- | --- | --- | --- | --- |
|  | Intervention | Control | Intervention | Control | Intervention | Control |
| Bihar |  |  |  |  |  |  |
| Attended AHD/VHSND | 27 | 3.4 | 48.4 | 37.6 | 52.9 | 44.6 |
| Attended PLA meeting | 28.7 | - | 32.2 | - | 37.2 | - |
| Attended AHD/VHSND  and PLA meeting | 16.9 | - | 26 | - | 31.8 | - |
| No meeting | 57.7 | 96.6 | 40.2 | 62.4 | 38.1 | 55.4 |
| Chhattisgarh |  |  |  |  |  |  |
| Attended AHD/VHSND | 46.8 | 18.1 | 49 | 32.6 | 54.4 | 33.3 |
| Attended PLA meeting | 25.7 | - | 37.6 | - | 37.3 | - |
| Attended AHD/VHSND  and PLA meeting | 23.9 | - | 32.7 | - | 34.2 | - |
| No meeting | 48.8 | 81.9 | 42.8 | 67.4 | 39.7 | 66.7 |
| Odisha |  |  |  |  |  |  |
| Attended AHD/VHSND | 40.4 | 23.8 | 66.9 | 59.7 | 75.8 | 60.6 |
| Attended PLA meeting | 26.6 | - | 25.6 | - | 36.9 | - |
| Attended AHD/VHSND  and PLA meeting | 16.3 | - | 23.6 | - | 34.8 | - |
| No meeting | 49.1 | 76.2 | 29.3 | 40.3 | 20.2 | 39.4 |

Note: The figures here represent the participation in AHD/VHSND and PLA meetings in the control and intervention areas of the selected blocks of Bihar, Chhattisgarh and Odish
