# Supplementary material for: Evaluation of impact of engaging federations of women groups to improve women’s nutrition interventions- before, during and after pregnancy in social and economically backward geographies: Evidence from three eastern Indian States
Source: PLoS One. 2023 Oct 5;18(10):e0291866. doi: 10.1371/journal.pone.0291866 (PMC10553280; doi:10.1371/journal.pone.0291866)
Supplement: S4 Table — (DOCX) [file pone.0291866.s006.docx]

**Table S4: Access to Nutrition Specific and nutrition sensitive intervention package for adolescent girls age 10-19 years in intervention and control area by participation status in AHD and PLA meeting**

|  | Bihar | | | | | Chhattisgarh | | | | | Odisha | | | | |
| --- | --- | --- | --- | --- | --- | --- | --- | --- | --- | --- | --- | --- | --- | --- | --- |
|  | Intervention | | Control | |  | Intervention | | Control | |  | Intervention | | Control | |  |
|  | Baseline 2016 | Endline 2021 | Baseline 2016 | Endline 2021 | DID | Baseline 2016 | Endline 2021 | Baseline 2016 | Endline 2021 | DID | Baseline 2016 | Endline 2021 | Baseline 2016 | Endline 2021 | DID |
| N | **863** | 567 | 841 | 552 |  | 1468 | 1053 | 1453 | 1025 |  | 655 | 517 | 970 | 527 |  |
| Improve food and nutrient intake |  |  |  |  |  |  |  |  |  |  |  |  |  |  |  |
| Minimum dietary diversity (6 out of 10 food groups) (%) | 12.3 | 50 | 14.2 | 37.5 | 14.34*** | 13.6 | 40.8 | 13.9 | 37.7 | 3.36 | 33.6 | 28 | 25.6 | 35.7 | -15.72*** |
| Living in a household with iodized salt (%) | 78.8 | 96.7 | 68 | 96.2 | -10.28*** | 95.3 | 97.8 | 94.7 | 97.5 | -0.33 | 45.3 | 99.8 | 56.3 | 99.3 | 11.47*** |
| Living in households with a kitchen garden (%) | 28.2 | 43 | 18.2 | 39.1 | -6.01 | 50 | 54.5 | 33.2 | 33.5 | 4.16 | 47.9 | 59.5 | 54.7 | 61.2 | 5.13 |
| Increase access to education sanitation and commodities for WASH |  |  |  |  |  |  |  |  |  |  |  |  |  |  |  |
| Living in households which do not practice open defecation (%) | 25.5 | 62.7 | 15.7 | 72.4 | -19.53*** | 14.8 | 71.6 | 18.9 | 74.1 | 1.54 | 16.5 | 42.3 | 17.2 | 35.6 | 7.48* |
| Percentage of using safe pads or sanitary pads | 30.3 | 74.7 | 26.2 | 57 | 13.66** | 36.1 | 61 | 37.3 | 59.5 | 2.70 | 48.7 | 85.9 | 46.8 | 82.5 | 1.55 |
| Prevent micronutrient deficiencies and anaemia |  |  |  |  |  |  |  |  |  |  |  |  |  |  |  |
| Consumed 4 or more IFA tablets (%) | 3.2 | 11.1 | 2.1 | 7.6 | 2.40 | 10.1 | 10.2 | 12 | 10.1 | 2.08 | 13.6 | 31 | 19.7 | 33.6 | 3.52 |
| Consumed deworming tablets (%) | 41.5 | 91.5 | 35.2 | 87.7 | -2.49 | 58 | 60.9 | 65.6 | 54.4 | 13.99*** | 33.7 | 84.9 | 34 | 77.4 | 7.84* |
| Increase access to health services and special care to nutritionally ‘at-risk’ adolescent (BMI<18.25) |  |  |  |  |  |  |  |  |  |  |  |  |  |  |  |
| Adolescent girls who visit Anganwadi Centre (AWC) for any service (%) | 3.2 | 43.9 | 3.6 | 31 | 13.19*** | 32.3 | 45.8 | 41.9 | 34.6 | 20.76*** | 24.4 | 35.1 | 30 | 32.2 | 8.45* |
| Nutritional Status |  |  |  |  |  |  |  |  |  |  |  |  |  |  |  |
| Thin (BMI<18.5) (%) | 24.7 | 18.2 | 21.8 | 17.3 | -1.95 | 15.7 | 12 | 12.9 | 10.4 | -1.17 | 12.1 | 9 | 12.6 | 8.4 | 1.13 |
