# Supplementary material for: Evaluation of impact of engaging federations of women groups to improve women’s nutrition interventions- before, during and after pregnancy in social and economically backward geographies: Evidence from three eastern Indian States
Source: PLoS One. 2023 Oct 5;18(10):e0291866. doi: 10.1371/journal.pone.0291866 (PMC10553280; doi:10.1371/journal.pone.0291866)
Supplement: S5 Table — (DOCX) [file pone.0291866.s007.docx]

**Table S5: Access to Nutrition Specific and nutrition sensitive intervention package among pregnant women, Swabhimaan Program in intervention and control area by participation status in VHSND and PLA meeting**

|  | Bihar | | | | | Chhattisgarh | | | | | Odisha | | | | | |
| --- | --- | --- | --- | --- | --- | --- | --- | --- | --- | --- | --- | --- | --- | --- | --- | --- |
|  | Intervention | | Control | |  | Intervention | | Control | |  | Intervention | | Control | |  |  |
|  | Baseline 2016 | Endline 2021 | Baseline 2016 | Endline 2021 | DID | Baseline 2016 | Endline 2021 | Baseline 2016 | Endline 2021 | DID | Baseline 2016 | Endline 2021 | Baseline 2016 | Endline 2021 | DID |  |
| N | 468 | 212 | 468 | 231 |  | 442 | 362 | 381 | 353 |  | 367 | 242 | 447 | 298 |  |  |
| Improve food and nutrient intake |  |  |  |  |  |  |  |  |  |  |  |  |  |  |  |  |
| Minimum dietary diversity (6 out of 10 food groups) (%) | 13.6 | 52.8 | 16.6 | 42.2 | 13.64* | 24.7 | 41.1 | 24.9 | 35.6 | 5.68 | 24.1 | 35.9 | 23.6 | 35.3 | 0.1 |  |
| Living in a household with iodized salt (%) | 79.9 | 97.9 | 66.5 | 94.6 | -10.09** | 97.1 | 99.2 | 96.6 | 97.8 | 0.97 | 57.5 | 99.6 | 61.1 | 100 | 3.17 |  |
| Living in food secure households (%) | 24.4 | 22.7 | 25 | 26.2 | -2.86 | 40.7 | 42.4 | 38.7 | 44.2 | -3.81 | 0 | 43.2 | 0 | 41.6 | 1.54 |  |
| Living in households with a kitchen garden (%) | 18.8 | 41.2 | 14.3 | 31.8 | 4.92 | 52.3 | 55.3 | 30.7 | 36.1 | -2.37 | 38.1 | 56.6 | 49 | 55.4 | 12.09* |  |
| Received ICDS entitlement for  supplementary food in month preceding survey (%) | 16.2 | 34.2 | 26.1 | 25.6 | 18.49*** | 38.9 | 82.8 | 39.1 | 81.9 | 1.08 | 30.5 | 84.7 | 35 | 82.6 | 6.64 |  |
| Increase access to education and commodities for WASH |  |  |  |  |  |  |  |  |  |  |  |  |  |  |  |  |
| Living in households which do not practice open defecation (%) | 21.4 | 60.7 | 8.8 | 64.6 | -16.50** | 5.4 | 64.2 | 17.6 | 71.7 | 4.61 | 23.4 | 38.4 | 13.4 | 37.2 | -8.83 |  |
| Prevent micronutrient deficiencies and anemia |  |  |  |  |  |  |  |  |  |  |  |  |  |  |  |  |
| Consumed 25 or more IFA tablets in second third trimester (%) | 42.3 | 62.9 | 51 | 56.7 | 14.97 | 47.3 | 65.1 | 58.8 | 72.3 | 4.32 | 0 | 64.9 | 0 | 61.7 | 3.13 |  |
| Two calcium tablets in second trimester (%) | 11.8 | 63.1 | 13.7 | 46.6 | 18.43** | 0.2 | 61.6 | 0.8 | 67.4 | -5.22 | 42.8 | 61.6 | 46.3 | 53.7 | 11.41* |  |
| Prevent early, poorly spaced or unwanted pregnancies |  |  |  |  |  |  |  |  |  |  |  |  |  |  |  |  |
| Using a modern family planning method before current pregnancy(%) | 2.1 | 20.1 | 1.1 | 8.4 | 10.67** | 6.6 | 13.3 | 9.2 | 15.9 | 0.01 | 12.6 | 16.9 | 11.2 | 17.1 | -1.6 |  |
| Taking decisions about their own health care (%) | 59.4 | 74.7 | 55.3 | 68.8 | 1.85 | 71.4 | 70 | 75.1 | 68.1 | 5.55 | 90.5 | 78.1 | 85.2 | 73.5 | -0.62 |  |
| Taking decisions about making major purchases for the household (%) | 51.5 | 73.8 | 54.1 | 67.8 | 8.55 | 74.5 | 69.2 | 72.7 | 68.4 | -1.07 | 92.6 | 78.9 | 92.2 | 74.8 | 3.62 |  |
| Taking decisions about visits to family members or relatives (%) | 40 | 71.7 | 34.8 | 64.7 | 1.82 | 73.2 | 69.5 | 75.1 | 68.2 | 3.22 | 89.9 | 76 | 92.2 | 73.5 | 4.79 |  |
| Increase access to health services and special care to nutritionally ‘at-risk’ women (MUAC <23cm) |  |  |  |  |  |  |  |  |  |  |  |  |  |  |  |  |
| First antenatal checkup in first trimester (%) | 28.8 | 53.5 | 29.5 | 34.6 | 19.49** | 28.1 | 70.4 | 42 | 69.2 | 15.15** | 72.5 | 84.7 | 72 | 88.9 | -4.66 |  |
| Weighed at least once in first trimester (%) | 62 | 78.1 | 65.8 | 58.4 | 23.56*** | 73.6 | 92 | 88.8 | 94.2 | 13.00*** | 88.2 | 88 | 85.3 | 90.9 | -5.80 |  |
| Nutritional Status |  |  |  |  |  |  |  |  |  |  |  |  |  |  |  |  |
| MUAC<23 cm | 50.2 | 48.9 | 57.3 | 42.9 | 13.01* | 38.2 | 33.8 | 38.8 | 30.2 | 4.21 | 32.7 | 24 | 37.6 | 23.8 | 5.03 |  |
