# Supplementary material for: Evaluation of impact of engaging federations of women groups to improve women’s nutrition interventions- before, during and after pregnancy in social and economically backward geographies: Evidence from three eastern Indian States
Source: PLoS One. 2023 Oct 5;18(10):e0291866. doi: 10.1371/journal.pone.0291866 (PMC10553280; doi:10.1371/journal.pone.0291866)
Supplement: S6 Table — (DOCX) [file pone.0291866.s008.docx]

**Table S6: Access to Nutrition Specific and nutrition sensitive intervention package among mothers with children under 2 years of age in intervention and control areas by participation status in VHSND and PLA meeting**

|  | Bihar | | | | | Chhattisgarh | | | | | | | | Odisha | | | | | | |
| --- | --- | --- | --- | --- | --- | --- | --- | --- | --- | --- | --- | --- | --- | --- | --- | --- | --- | --- | --- | --- |
|  | Intervention | | Control | |  | Intervention | | Control | | |  | | Intervention | | | Control | | |  | |
|  | Baseline 2016 | Endline 2021 | Baseline 2016 | Endline 2021 | DID | Baseline 2016 | Endline 2021 | | Baseline 2016 | Endline 2021 | | DID | | Baseline 2016 | Endline 2021 | | Baseline 2016 | Endline 2021 | | DID |
| N | **1400** | 564 | 1212 | 598 |  | 1281 | 992 | | 1258 | 1090 | |  | | 1760 | 700 | | 1844 | 822 | |  |
| Improve food and nutrient intake |  |  |  |  |  |  |  | |  |  | |  | |  |  | |  |  | |  |
| Minimum dietary diversity (6 out of 10 groups) (%) | 9.2 | 50 | 12.6 | 41.9 | 11.47** | 18.8 | 43 | | 19.1 | 37.7 | | 5.68 | | 28.1 | 36.9 | | 21.4 | 39.3 | | -9.11** |
| Living in a household with iodized salt (%) | 82.3 | 98.4 | 71 | 94.6 | -7.52*** | 93.9 | 97.8 | | 93.6 | 98.3 | | -0.79 | | 57.3 | 99.4 | | 63.5 | 99.8 | | 5.74*** |
| Living in food secure households (%) | 20.4 | 18.8 | 22.6 | 18.8 | 2.09 | 28.3 | 36.9 | | 34.3 | 41 | | 1.93 | | 19 | 36.3 | | 12.6 | 32.6 | | -2.74 |
| Living in households with a kitchen garden (%) | 16.2 | 44.3 | 14.2 | 35.3 | 6.95* | 48.9 | 54.8 | | 26.1 | 30.2 | | 1.74 | | 33.5 | 58.8 | | 42.7 | 50.7 | | 17.3*** |
| Received minimum PDS in month preceding survey (%) | 64.2 | 98 | 72.5 | 96.4 | 9.97*** | 97.5 | 98.3 | | 96.2 | 98.2 | | -1.16 | | 55.7 | 85.7 | | 60.4 | 85.8 | | 4.55 |
| Received ICDS entitlement for supplementary food  in month preceding survey (%) | 17.3 | 54.4 | 27.8 | 49.8 | 15.15*** | 43.8 | 91.5 | | 41.9 | 92.1 | | -2.54 | | 66.7 | 99.4 | | 69.4 | 99.7 | | 2.31 |
| Increase access to education/commodities for WASH |  |  |  |  |  |  |  | |  |  | |  | |  |  | |  |  | |  |
| Living in households which do not use safe sanitation services (%) | 20.4 | 65.5 | 9.3 | 64.6 | -10.29** | 16.1 | 65.5 | | 17.6 | 70.4 | | -3.42 | | 21.7 | 40.7 | | 15.4 | 31.1 | | 3.28 |
| Prevent micronutrient deficiencies and anaemia |  |  |  |  |  |  |  | |  |  | |  | |  |  | |  |  | |  |
| Consumed 100 or more IFA tablets  during last pregnancy (%) | 10.7 | 27 | 14.2 | 15.6 | 14.85*** | 17.4 | 29.7 | | 27 | 35.5 | | 3.65 | | 30.6 | 66.6 | | 42.5 | 71.9 | | 6.64* |
| Consumed 100 or more calcium tablets  during last pregnancy (%) | 0.4 | 22.7 | 0.2 | 13.2 | 9.40*** | 0 | 18.5 | | 0 | 22.9 | | -4.34* | | 0 | 49.2 | | 0.1 | 49.4 | | -0.14 |
| Prevent early, poorly spaced or unwanted pregnancies |  |  |  |  |  |  |  | |  |  | |  | |  |  | |  |  | |  |
| Using a modern family planning method (%) | 7.9 | 24.7 | 6.6 | 14.9 | 8.58** | 5.5 | 33.5 | | 9.5 | 32.4 | | 5.19* | | 22.6 | 35.7 | | 20.7 | 25.6 | | 8.16** |
| Taking decisions about their own health care (%) | 59.3 | 70.8 | 66.7 | 69.6 | 8.64* | 68.1 | 60.5 | | 68.4 | 68.7 | | -8.06** | | 72.5 | 87.4 | | 73 | 87.6 | | 0.39 |
| Taking decisions about making major purchases  for the household (%) | 55.1 | 70.3 | 62.5 | 68.7 | 8.89* | 74.2 | 60.8 | | 73.8 | 68.6 | | -8.28** | | 75.5 | 87.1 | | 76.8 | 84.2 | | 4.28 |
| Taking decisions about visits to  family members or relatives (%) | 43.4 | 69.1 | 48.5 | 68.7 | 5.52 | 72.7 | 59.7 | | 72.5 | 68.4 | | -8.91** | | 77.3 | 88.3 | | 81.6 | 87.5 | | 5.10* |
| Increase access to health services and special care  to nutritionally ‘at-risk’ women (MUAC <23cm) |  |  |  |  |  |  |  | |  |  | |  | |  |  | |  |  | |  |
| First antenatal checkup in first trimester (%) | 36.3 | 56.1 | 27.7 | 42.7 | 4.89 | 27.4 | 69.5 | | 35.4 | 68.3 | | 9.25*** | | 27.2 | 72.4 | | 18.2 | 72 | | -8.67** |
| Received at least 4 antenatal care (%) | 16.4 | 31.8 | 14.5 | 18.3 | 11.54*** | 15.8 | 45.9 | | 31.7 | 52 | | 9.82*** | | 21.4 | 57 | | 17.4 | 56.2 | | -3.14 |
| Weighed at least four times in last pregnancy (%) | 6.2 | 20.8 | 3.5 | 8.8 | 9.25*** | 18 | 32.6 | | 27.3 | 37.8 | | 4.03 | | 38.3 | 63.4 | | 37 | 56.7 | | 5.43 |
| Accessed JSY | 58.8 | 55.6 | 59.5 | 39.4 | 16.96*** | 47.7 | 46.4 | | 47.8 | 54.5 | | -7.96** | | 49.1 | 59.7 | | 53 | 54.3 | | 9.29** |
| Delivered in a health facility in last pregnancy (%) | 78.9 | 90.2 | 76.2 | 75.2 | 12.35*** | 62.2 | 69.2 | | 67.2 | 72.5 | | 1.57 | | 76.9 | 90.6 | | 65.6 | 79.2 | | 0.42 |
| Nutritional Status |  |  |  |  |  |  |  | |  |  | |  | |  |  | |  |  | |  |
| Mothers with height<145 cm (%) | 18.1 | 10.2 | 14.8 | 9.4 | -2.63 | 62.2 | 10.2 | | 67.2 | 7.2 | | 0.00 | | 14.7 | 14.3 | | 16.1 | 13.1 | | 2.48 |
| Mothers who are thin (BMI<18.5) (%) | 44.8 | 36.3 | 45 | 37.3 | -0.75 | 53.6 | 42.5 | | 54.6 | 44.1 | | -0.52 | | 45.6 | 36.3 | | 45.3 | 35.2 | | 0.94 |
