# Supplementary material for: Evaluation of impact of engaging federations of women groups to improve women’s nutrition interventions- before, during and after pregnancy in social and economically backward geographies: Evidence from three eastern Indian States
Source: PLoS One. 2023 Oct 5;18(10):e0291866. doi: 10.1371/journal.pone.0291866 (PMC10553280; doi:10.1371/journal.pone.0291866)
Supplement: S7 Table — (DOCX) [file pone.0291866.s009.docx]

**Table S7: Access to Nutrition Specific and nutrition sensitive intervention package for adolescent girls age 10-19 years in intervention area by participation status and frequency of participation in AHD and PLA meeting**

|  |  | Intervention | | |  | Intervention | | |
| --- | --- | --- | --- | --- | --- | --- | --- | --- |
|  |  | AHD | | |  | PLA meeting | | |
|  | Attended AHD | No contacts | 1-5 contacts | 6 or more contacts | Attended PLA meeting | No contacts | 1-5 contacts | 6 or more contacts |
| N | 814 | 1323 | 615 | 194 | 577 | 1560 | 340 | 235 |
| Improve food and nutrient intake |  |  |  |  |  |  |  |  |
| Minimum dietary diversity (6 out of 10 food groups) (%) | 40 | 38.1 | 38.1 | 45.4 | 39.1 | 38.7 | 39.5 | 38.6 |
| Living in a household with iodized salt (%) | 98.3 | 98 | 98.7 | 97 | 98.3 | 98.1 | 98.7 | 97.8 |
| Living in households with a kitchen garden (%) | 64.7 | 44.9 | 62.9 | 70.3 | 67 | 47 | 68.7 | 64.5 |
| Increase access to education sanitation and commodities for WASH |  |  |  |  |  |  |  |  |
| Living in households which do not practice open defecation (%) | 65.6 | 54.5 | 62.6 | 75.1 | 65.2 | 56.3 | 62 | 69.8 |
| Percentage of using safe pads or sanitary pads | 79.2 | 67.6 | 77.9 | 83.6 | 79.9 | 69.3 | 77.7 | 83.3 |
| Prevent micronutrient deficiencies and anaemia |  |  |  |  |  |  |  |  |
| Consumed 4 or more IFA tablets (%) | 23.2 | 14.1 | 22.1 | 26.4 | 24.7 | 14.9 | 21.7 | 28.9 |
| Consumed deworming tablets (%) | 77.5 | 73.9 | 78.7 | 73.7 | 79.3 | 73.8 | 78.6 | 80.3 |
| Increase access to health services and special care to nutritionally ‘at-risk’ adolescent (BMI<18.25) |  |  |  |  |  |  |  |  |
| Adolescent girls who visit Anganwadi Centre (AWC) for any service (%) | 62.9 | 28.3 | 63.5 | 61.1 | 61.6 | 34.1 | 60.9 | 62.7 |
| Nutritional Status |  |  |  |  |  |  |  |  |
| Thin (BMI<18.5) (%) | 10.1 | 14.7 | 10.9 | 7.7 | 10.1 | 14 | 11.6 | 7.9 |
| Experiencing double burden of short and thin (%) | 3.3 | 4 | 4 | 0.9 | 3 | 4 | 2.9 | 3.1 |

Note: Difference-in-difference here refers to change in outcome over time between intervention and Control area
