# Supplementary material for: Evaluation of impact of engaging federations of women groups to improve women’s nutrition interventions- before, during and after pregnancy in social and economically backward geographies: Evidence from three eastern Indian States
Source: PLoS One. 2023 Oct 5;18(10):e0291866. doi: 10.1371/journal.pone.0291866 (PMC10553280; doi:10.1371/journal.pone.0291866)
Supplement: S8 Table — (DOCX) [file pone.0291866.s010.docx]

**Table S8: Access to Nutrition Specific and nutrition sensitive intervention package among pregnant women in intervention area by participation status and frequency of participation in VHSND and PLA meeting**

|  |  |  | VHSND |  |  |  | PLA meeting |  |
| --- | --- | --- | --- | --- | --- | --- | --- | --- |
|  | Attended VHSND | No | 1 to 5 | 6 or more contacts | Attended PLA meeting | No | 1 to 5 | 6 or more contacts |
|  | 433 | 383 | 313 | 122 | 267 | 549 | 202 | 65 |
| Improve food and nutrient intake |  |  |  |  |  |  |  |  |
| Minimum dietary diversity (6 out of 10 food groups) (%) | 50.8 | 37.2 | 50.1 | 52.4 | 55 | 39.3 | 53.2 | 59.7 |
| Living in a household with iodized salt (%) | 98.6 | 99.1 | 98.9 | 97.6 | 99.1 | 98.7 | 98.8 | 100 |
| Living in food secure households (%) | 35.1 | 35.2 | 35.5 | 34.2 | 37.4 | 34.1 | 40.2 | 28.7 |
| Living in households with a kitchen garden (%) | 63.3 | 35.6 | 62.9 | 64.5 | 66.3 | 42.6 | 66 | 67.3 |
| Received ICDS entitlement for supplementary food in month preceding survey (%) | 71.4 | 57.9 | 73.8 | 65.2 | 67.7 | 63.8 | 74.6 | 46 |
| Increase access to education and commodities for WASH |  |  |  |  |  |  |  |  |
| Living in households which do not practice open defecation (%) | 60.6 | 52.2 | 63.7 | 52.7 | 62.4 | 53.9 | 64.5 | 55.7 |
| Prevent micronutrient deficiencies and anaemia |  |  |  |  |  |  |  |  |
| Consumed 25 or more IFA tablets in second third trimester (%) | 68.4 | 59.6 | 65.2 | 76.5 | 67.4 | 62.9 | 63.6 | 80.9 |
| Two calcium tablets in second trimester (%) | 70.6 | 52.4 | 67.3 | 80 | 74.3 | 56.3 | 73.5 | 77.2 |
| Prevent early, poorly spaced or unwanted pregnancies |  |  |  |  |  |  |  |  |
| Using a modern family planning method before current pregnancy(%) | 20.9 | 12.1 | 17.4 | 29.7 | 21.7 | 14.3 | 20.1 | 26.9 |
| Taking decisions about their own health care (%) | 68.1 | 80 | 66.5 | 72.2 | 65.6 | 77.6 | 66.4 | 62.9 |
| Taking decisions about making major purchases for the household (%) | 68 | 79.3 | 66 | 73.1 | 64 | 77.8 | 63.1 | 66.8 |
| Taking decisions about visits to family members or relatives (%) | 65.8 | 78.8 | 64.5 | 68.9 | 64 | 75.7 | 64.9 | 60.9 |
| Increase access to health services and special care to nutritionally ‘at-risk’ women (MUAC <23cm) |  |  |  |  |  |  |  |  |
| First antenatal checkup in first trimester (%) | 76.5 | 57.3 | 74.4 | 81.8 | 77.5 | 62.6 | 78.8 | 73.5 |
| Weighed at least once in first trimester (%) | 91.7 | 79.2 | 93.6 | 86.9 | 88.9 | 84.3 | 90.5 | 84 |
| Height was recorded (%) | 39.3 | 22.7 | 43.6 | 28.4 | 45.6 | 24.7 | 48.9 | 35.1 |
| Nutritional Status |  |  |  |  |  |  |  |  |
| MUAC<23 cm | 37.2 | 37 | 33.9 | 45.8 | 36 | 37.6 | 36 | 36 |
