# Supplementary material for: Evaluation of impact of engaging federations of women groups to improve women’s nutrition interventions- before, during and after pregnancy in social and economically backward geographies: Evidence from three eastern Indian States
Source: PLoS One. 2023 Oct 5;18(10):e0291866. doi: 10.1371/journal.pone.0291866 (PMC10553280; doi:10.1371/journal.pone.0291866)
Supplement: S9 Table — (DOCX) [file pone.0291866.s011.docx]

**Table S9: Access to Nutrition Specific and nutrition sensitive intervention package among mothers with children under 2 years of age in intervention area by participation status and frequency of participation in VHSND and PLA meeting**

|  |  | Intervention | | |  | Intervention | | |
| --- | --- | --- | --- | --- | --- | --- | --- | --- |
|  |  | VHSND | | |  | PLA meeting | | |
|  | Attended VHSND | No contacts | 1-5 contacts | 6 or more contacts | Attended PLA meeting | No contacts | 1-5 contacts | 6 or more contacts |
| N | 1347 | 909 | 808 | 539 | 837 | 1419 | 562 | 277 |
| Improve food and nutrient intake |  |  |  |  |  |  |  |  |
| Minimum dietary diversity (6 out of 10 food groups) (%) | 47.9 | 38.3 | 47.6 | 48.2 | 48.2 | 41.7 | 48.2 | 48.3 |
| Living in a household with iodized salt (%) | 98.9 | 97.8 | 98.7 | 99 | 99.3 | 98 | 99.7 | 98.3 |
| Living in food secure households (%) | 29.1 | 29.7 | 28.6 | 29.9 | 30.4 | 28.8 | 33.2 | 24.6 |
| Living in households with a kitchen garden (%) | 58.8 | 41 | 53.4 | 66.9 | 66.5 | 42.9 | 64.1 | 71.3 |
| Received minimum PDS entitlement in month preceding survey (%) | 93.3 | 94.7 | 95 | 90.7 | 95.8 | 92.6 | 95.2 | 96.9 |
| Received ICDS entitlement for supplementary food in month preceding survey (%) | 82.8 | 72.3 | 84 | 81.2 | 82.6 | 76.2 | 87.5 | 72.7 |
| Increase access to education and commodities for WASH |  |  |  |  |  |  |  |  |
| Living in households which do not practice open defecation (%) | 57.7 | 60 | 60.1 | 54.2 | 62.5 | 56.3 | 62.4 | 62.8 |
| Prevent micronutrient deficiencies and anaemia |  |  |  |  |  |  |  |  |
| Consumed 100 or more IFA tablets during last pregnancy (%) | 43.7 | 31.1 | 33.1 | 58.8 | 47.3 | 33.5 | 43.5 | 54.7 |
| Consumed 100 or more calcium tablets during last pregnancy (%) | 31.6 | 24.3 | 21.9 | 46 | 34.8 | 25.1 | 29.1 | 46.2 |
| Prevent early, poorly spaced or unwanted pregnancies |  |  |  |  |  |  |  |  |
| Using a modern family planning method (%) | 34.4 | 24.8 | 29.3 | 42.1 | 39.8 | 25.1 | 32.7 | 54.2 |
| Taking decisions about their own health care (%) | 71.3 | 73.2 | 66.6 | 78.5 | 70.7 | 72.9 | 70.5 | 71.3 |
| Taking decisions about making major purchases for the household (%) | 71.2 | 73 | 66.1 | 78.8 | 69.3 | 73.4 | 68.5 | 70.9 |
| Taking decisions about visits to family members or relatives (%) | 70.4 | 72.9 | 67 | 75.4 | 70.4 | 72 | 71.7 | 67.7 |
| Increase access to health services and special care to nutritionally ‘at-risk’ women (MUAC <23cm) |  |  |  |  |  |  |  |  |
| First antenatal checkup in first trimester (%) | 69.9 | 57.4 | 67.8 | 73 | 69.3 | 62.3 | 67.3 | 73.2 |
| Received at least 4 antenatal care in last pregnancy (%) | 46.7 | 38.2 | 35.5 | 63.4 | 52.3 | 37.9 | 46.3 | 64.3 |
| Height was recorded (%) | 42.5 | 32.9 | 50 | 31.3 | 50.5 | 31.6 | 53.3 | 44.9 |
| Weighed at least four times in last pregnancy (%) | 40.3 | 30.4 | 27.3 | 59.8 | 40.9 | 33.6 | 35.1 | 52.4 |
| Accessed JSY | 56.1 | 50.3 | 49.6 | 66 | 58.5 | 51 | 57.1 | 61.2 |
| Delivered in a health facility in last pregnancy (%) | 83.7 | 83.7 | 80.4 | 88.6 | 82.5 | 84.5 | 80.1 | 87.3 |
| Nutritional Status |  |  |  |  |  |  |  |  |
| Mothers who are thin (BMI<18.5) (%) | 37.7 | 39.2 | 40 | 34.4 | 38.8 | 38 | 37.7 | 41.1 |
