# Supplementary material for: Evaluation of impact of engaging federations of women groups to improve women’s nutrition interventions- before, during and after pregnancy in social and economically backward geographies: Evidence from three eastern Indian States
Source: PLoS One. 2023 Oct 5;18(10):e0291866. doi: 10.1371/journal.pone.0291866 (PMC10553280; doi:10.1371/journal.pone.0291866)
Supplement: S10 Table — (DOCX) [file pone.0291866.s012.docx]

**Table S10: Multivariable logistic regression of association between receipt of nutrition specific services and nutrition outcomes with frequency of participation in AHD or VHSND adjusted for socio-economic characteristics, Endline Survey, 2021**

|  | Adolescent Girls | | | | Pregnant women | | | | Mother of children under 2 years | | | |
| --- | --- | --- | --- | --- | --- | --- | --- | --- | --- | --- | --- | --- |
|  | 1 to 5 contacts | | 6+ contacts | | 1 to 5 contacts | | 6+ contacts | | 1 to 5 contacts | | 6+ contacts | |
| Improve food and nutrient intake | |  | |  | |  | |  | |  | |  |
| Minimum dietary diversity (6 out of 10 food groups) | 1.18 | | 0.89 | | 1.80** | | 2.35** | | 1.36* | | 1.21 | |
| Living in a household with iodized salt | 1.23 | | 0.78 | | 1.11 | | 1 | | 7.13** | | 1.27 | |
| Living in food secure households |  | |  | | 1.3 | | 0.78 | | 1.1 | | 0.79 | |
| Living in households with a kitchen garden | 2.50*** | | 2.01*** | | 2.70*** | | 3.21*** | | 2.15*** | | 3.40*** | |
| Received minimum PDS entitlement in month preceding survey |  | |  | |  | |  | | 1.4 | | 2.18* | |
| Received ICDS entitlement for supplementary food in month preceding survey | 3.20*** | | 3.18*** | | 1.43 | | 0.45** | | 1.70** | | 0.85 | |
| Increase access to education sanitation and commodities for WASH | |  | |  | |  | |  | |  | |  |
| Living in households which do not practice open defecation | 1.36* | | 1.63** | | 1.57* | | 0.97 | | 1.19 | | 1.12 | |
| Percentage of using safe pads or sanitary pads | 1.57** | | 2.20*** | |  | |  | |  | |  | |
| Consumed IFA tablets | 1.55* | | 2.38*** | | 1 | | 2.43* | | 1.45** | | 2.59*** | |
| Consumed calcium tablets |  | |  | | 2.15*** | | 2.39* | | 1.2 | | 2.62*** | |
| Consumed deworming tablets (%) | 1.94*** | | 2.31*** | |  | |  | |  | |  | |
| Prevent early, poorly spaced or unwanted pregnancies | |  | |  | |  | |  | |  | |  |
| Using a modern family planning method |  | |  | | 1.49 | | 2.10* | | 1.29* | | 3.59*** | |
| Taking decisions about their own health care |  | |  | | 0.57** | | 0.47* | | 0.89 | | 0.96 | |
| Taking decisions about making major purchases for the household |  | |  | | 0.49*** | | 0.56 | | 0.79 | | 0.91 | |
| Taking decisions about visits to family members or relatives |  | |  | | 0.59** | | 0.49* | | 0.97 | | 0.83 | |
| Increase access to health services | |  | |  | |  | |  | |  | |  |
| First antenatal checkup in first trimester |  | |  | | 2.10*** | | 1.7 | | 1.13 | | 1.76** | |
| Received antenatal care |  | |  | |  | |  | | 1.32* | | 3.29*** | |
| Height was recorded |  | |  | | 2.74*** | | 1.69 | | 2.26*** | | 1.73*** | |
| Weighed |  | |  | | 1.49 | | 0.98 | | 0.95 | | 2.40*** | |
| Accessed JSY |  | |  | |  | |  | | 1.34* | | 1.49* | |
| Delivered in a health facility in last pregnancy |  | |  | |  | |  | | 0.77* | | 1.25 | |
| Nutritional Status | |  | |  | |  | |  | |  | |  |
| Thin | 0.88 | | 0.55* | | 1.04 | | 0.87 | | 1.01 | | 1.21 | |

Note: Reference category is 0 contacts. Adjusted for socio-economic characteristics of the respondent such as age, education, wealth, social group and religion. . Level of significance ∗P < 0.10; ∗∗P < 0.05; ∗∗∗P < 0.01. Figures in parenthesis are 95% confidence interval.
